# The difference in endothelium‐dependent relaxation components in proximal and distal thoracic aorta regions of male rats

**DOI:** 10.14814/phy2.15992

**Published:** 2024-03-27

**Authors:** O. R. Mezhenskyi, I. B. Philyppov

**Affiliations:** ^1^ Department of Nervous and Muscular Physiology Bogomoletz Institute of Physiology Kiyv Ukraine

**Keywords:** aorta, differential expression, endothelium‐dependent relaxation, K_IR_ channel, smooth muscle cells

## Abstract

Aorta, the largest vessel in the body, is generally considered anatomically homogeneous, yet spatial functional differences exist. In our study, we conducted a comprehensive analysis by reexamining public RNA‐SEQ data, comparing expression patterns between thoracic and abdominal aorta. Additionally, we measured acetylcholine‐induced relaxations of the different regions of thoracic aorta in Wistar Rats. Our results revealed a distinct percentage difference in acetylcholine‐induced relaxation in the proximal and distal segments of the thoracic aorta (*p* = 1.14e‐4). To explain this variation, we performed differential expression analysis of previously published RNA‐sequencing data between thoracic and abdominal aorta, which showed 497 differentially expressed genes between these locations. From results of RNA‐Seq analysis, we draw a hypothesis that differential expressions of the potassium inward rectifying channels (K_IR_) and voltage gated calcium channels (VGCC) presumably located on SMC, with higher expression in the distal thoracic segments in comparison with the proximal thoracic segments of aorta, can explain differences in acetylcholine‐induced relaxation. Notably, specific blockade of K_IR_ eliminated differences between the proximal and distal regions of thoracic aorta, underscoring their significance in understanding the spatial nuances in aortic behavior, also blockade of VGCC, shows a higher effect on basal tone, in distal region of thoracic aorta in comparison with proximal.

## 1 INTRODUCTION

The aorta originates at the aortic arch near the heart, extending downward as the proximal thoracic aorta. It traverses the thoracic cavity, supplying oxygenated blood to organs within. The aorta's inherent elasticity allows it to expand and contract synchronously with each heartbeat, ensuring a continuous bloodstream. Additionally, the aorta functions as a buffer, absorbing pressure spikes to safeguard smaller vessels from damage and contribute to maintaining stable blood pressure levels (Shahoud et al., 2023; White et al., 2023).

The regulation of the aorta involves various mechanisms, with produced by eNOs, basal nitric oxide secretion standing out as one of the most critical factors in regulating basal tone (Laurindo et al., 2018). It is known that the production of nitric oxide can be regulated by exogenous acetylcholine, which acts as the main mediator of endothelium‐dependent relaxation.

Another mechanism connected to aorta regulation is the endothelium‐dependent hyperpolarizing factor (EDHF) which is important to smooth muscle cells (SMC) relaxation through hyperpolarization of the cellular membrane (Laurindo et al., 2018). Potassium inward rectifying channels (K_IR_) are an important element of aortic SMC relaxation amplifying the effects of EDHF produced by potassium SK/IK/BK channels located on the endothelial membrane, causing hyperpolarization by releasing potassium from the cell (Ahn et al., 2017; Jackson, 2017; Werner & Ledoux, 2014).

Despite its considerable length, the anatomical features of the aorta wall exhibit minimal variations from proximal to distal ends. The anatomical aorta can be split into thoracic and abdominal parts, with both of them belonging to an elastic type of vessel, but with a bigger population of SMC in the abdominal part (Shahoud et al., 2023).

Functional disparities in vascular behavior are discernible across species, with rats exemplifying distinctive properties in the physiological regulation of the different aorta regions. It was shown that abdominal vascular segment notably exhibits characteristics reminiscent of muscular arteries, yet intriguing nuances come to light. Particularly in response to acetylcholine, variations in endothelium‐dependent relaxations are observed, where abdominal regions manifest a heightened significance of EDHF compared to their thoracic counterparts and overall greater relaxation in response to acetylcholine. This nuanced responsiveness is accompanied by pronounced differences in stiffness, accentuating the complexity of vascular dynamics. Furthermore, an asymmetrical expression of connexins between these regions implies potential variations in the propagation of EDHF, unraveling the intricate tapestry of vascular regulation (Ameer et al., 2021; Guo et al., 2006; Honda et al., 1997; Ko et al., 2001; Lesauskaite et al., 2006; Oloyo et al., 2012; Reho et al., 2015; Ruddy et al., 2008; Zygmunt et al., 1995).

Also, some studies reveal that the total lumen volume and longitudinal stretch ratio increase from the upstream to downstream positions in the aorta, accompanied by a decrease in tension per lamellar unit, suggesting intrinsic variations in the vascular wall along the length of the aorta (Guo & Kassab, 2003; Wolinsky & Glagov, 1969).

Another component of aorta wall relaxation is connected to prostaglandins signaling, through activation of guanylate cyclase in SMC (Hristovska et al., 2007). Thus, there is no previous evidence of a spatial difference in prostaglandins effect on different aorta regions, but some studies show that when the effect of prostaglandins is abolished difference in behavior between aorta regions still exists, showing that other mechanisms should play a role in that phenomenon (Oloyo et al., 2012).

While the physiological roles of EDHF, prostaglandins, and nitric oxide in aorta relaxation are well established, certain ambiguities arise due to several reasons. The signaling pathways of the aforementioned factors can intersect, leading to a synergistic effect that makes the assessment of their individual roles challenging (Hristovska et al., 2007; Quyyumi & Ozkor, 2011). Another issue related to the contribution of these components is the slightly ambiguous results presented in different articles, which could be attributed to variations in functionalities between different regions of the aorta, or between individual animals (Chitaley & Webb, 2002; Wang et al., 2003; Wong & Vanhoutte, 2010; Woodman et al., 2000; Zhao et al., 2015).

Thus, aorta relaxation mechanisms are very complex, the question arises if endothelium‐dependent relaxation mechanisms work the same across all thoracic aorta lengths, and where lies the functional border between the thoracic and abdominal aorta.

The aim of this study was threefold: first, to investigate the existence of differences in acetylcholine‐induced relaxation among thoracic aortic segments in Wistar Rat; second, to analyze RNA sequence data to identify differential expression patterns between thoracic and abdominal aorta; and finally, to seek a plausible explanation for the observed differences in acetylcholine‐induced relaxation through the results derived from RNA‐SEQ analysis.

## 2 MATERIALS AND METHODS

### 2.1 Rat aorta smooth muscle strips preparation and contraction measurements

In our experiments, 3‐month‐old male Wistar rats were utilized as subjects, adhering to animal protocols in compliance with EU Directive 2010/63/EU for animal experiments (http://ec.europa.eu/environment/chemicals/lab_animals/legislation_en.htm). The experimental protocol received approval from the Bioethics Committee of the Bogomoletz Institute of Physiology (BIPh) under Permission No 2/17, granted on 05.09.2017. Rats used in the study were bred, housed, and cared for in the specialized animal facility (vivarium) of BIPh, with meticulous efforts to minimize any potential suffering.

**FIGURE 1:**
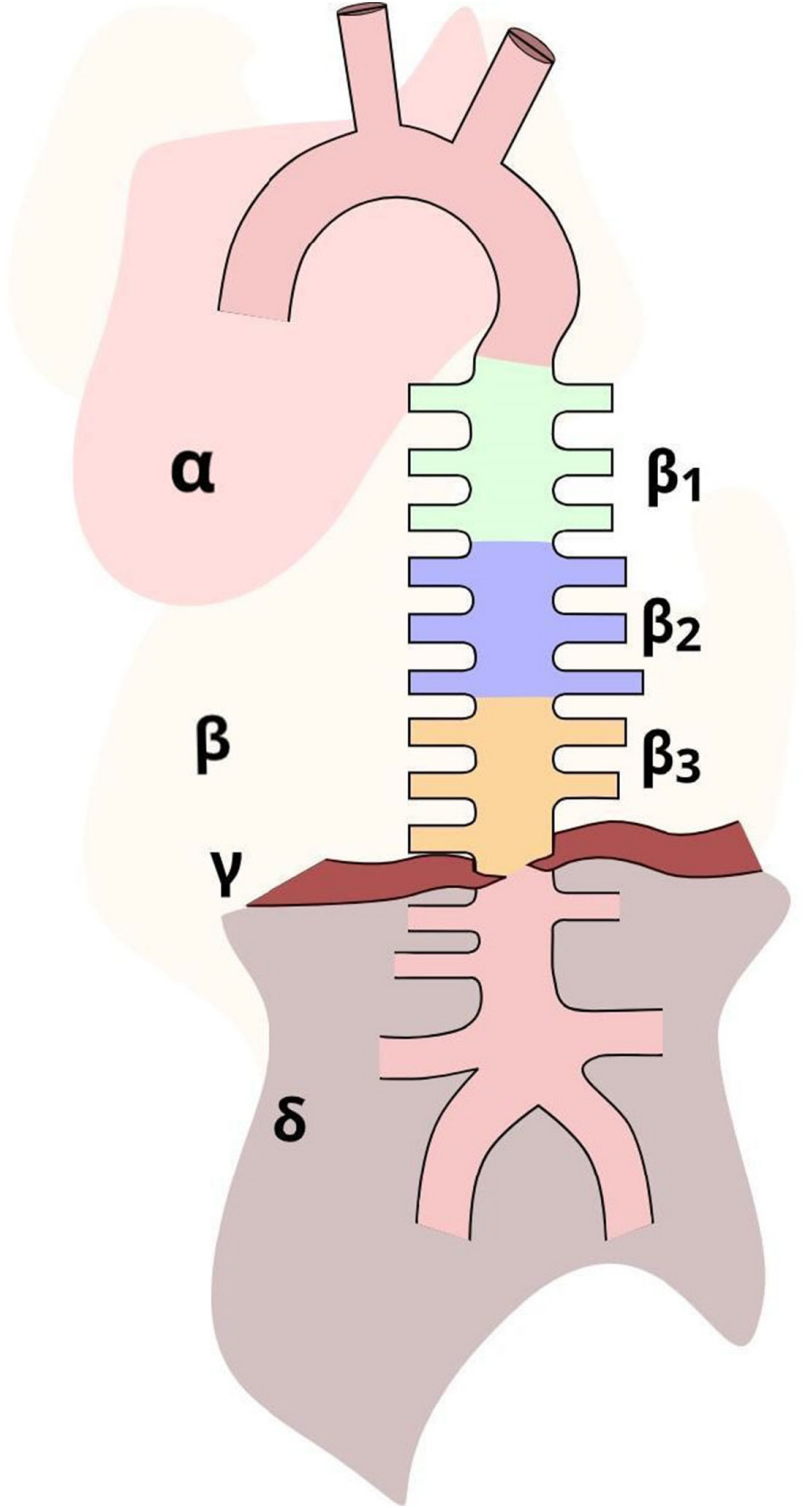
Anatomy of the aorta. The schematic anatomy of the aorta is depicted as follows: (*α*) aortic arch, (*β*) entire thoracic aorta from which intercostal arteries are branching, (*β*_1_) proximal region of the thoracic aorta, (*β*_2_) intermediate region of the thoracic aorta, (*β*_3_) distal region of the thoracic aorta, (*γ*) diaphragm, and (*δ*) abdominal aorta. The proximal, intermediate, and distal regions of the thoracic aorta were each cut into 5 mm rings, which were subsequently utilized for the measurement of their acetylcholine‐induced relaxation on phenylephrine preconstriction.

Male Wistar rats weighing between 200 and 250 g underwent euthanasia through exposure to a rising concentration of CO_2_, followed by confirmation of death through subsequent decapitation. In total, 30 rats were used in all experiments. Under stereo microscopic control, aortas were carefully dissected from the aortic arch to the renal arteries, with the removal of surrounding adipose tissues. In our experiments, six aortic segments each measuring approximately 0.5 cm in length were dissected based on the location of intercostal arterial branches using an eyepiece with a reticle, to ensure consistent length, categorizing them into three groups: the proximal segments of the thoracic aorta, intermediate segments of the thoracic aorta, and distal segments of the thoracic aorta (Figure 1).

To record the contraction and relaxation of aorta segments, they were suspended in a chamber filled with Krebs solution at a constant flow rate of 6 mL per minute. Krebs solution was composed of 120 mM of Na^+^, 5 mM of K^+^, 2 mM of Ca^+2^, 1.2 mM of Mg^+2^, 5 mM glucose, and 7 mM Hepes, and was balanced around 7.35 pH at 37°C. Each segment was secured by pairs of hooks—one hook firmly affixed to the chamber walls, while the other was attached to a self‐made tension sensor. Mechanical responses, measured isometrically, were converted to digital data by the Axon DigiData 1200 and displayed on a computer screen using Clampex 8.0.

Before initiating measurements, the strips were allowed to equilibrate in normal Krebs solution under a basal tension of 1 mN for 1 h. Specific tension values were calculated using the Mulvany and Halpern (1977) method, by calculations of passive wall‐tension internal circumference, which later were used to find out the necessary basal tension to obtain approximately 100 mm Hg of transmural pressure.

The viability of segments was tested using initial phenylephrine contraction and acetylcholine‐induced relaxation, where only rings with endothelium intact relaxed.

Submaximal tonic contraction of segments was induced by applying 10 μmol phenylephrine, and submaximal consistent relaxation was achieved by applying 1 μmol of acetylcholine, but not complete relaxation (90%–95%). Both concentrations were chosen based on previous experiments with building dose‐effect curves, which we don't include.

Data acquisition was performed using the Clampex software (Axon Instruments, USA), with subsequent analysis carried out in Clampfit (Axon Instruments, USA) and the R programming language (R Core Team, 2021).

Chemicals employed in the study include phenylephrine hydrochloride, acetylcholine chloride, barium chloride, ouabain octahydrate, nicardipine hydrochloride, and 18α‐glycyrrhetinic acid (Sigma‐Aldrich). All compounds were directly added from their respective stocks to the experimental Krebs solution to achieve the desired working concentrations which were as follows: 10 μmol for phenylephrine, 1 μmol for acetylcholine, 100 μmol for barium chloride, 40 μmol for oubaine, 1 μmol for nicardipine, and 10 μmol for 18α‐glycyrrhetinic acid (18GA) both dissolved in DMSO. Control experiments verified that DMSO at concentrations up to 0.1% did not impact the contractility of the aorta rings.

### 2.2 Analysis of RNA‐seq data

For the analysis of RNA‐seq data, we utilized the R package Deseq2 (Love et al., 2014) software, enabling a comprehensive examination of gene expression patterns and differential gene expression across the various experimental conditions.

Differential expressions were studied between the thoracic and abdominal regions of the rat aorta to find differences in expression profile gradient, which may be extended to explain dissimilarities in the proximal and distal (close to abdominal) ends of the thoracic aorta.

Raw data files were obtained from the Sequence Read Archive (SRA) and subsequently aligned to the reference genome, with quantification performed using the hisat2 software (Zhang et al., 2021). Sequence data were aligned to the reference genome—mRatBN7.2 and then converted to count data. In total, two samples from the thoracic aorta (Reho et al., 2015) and five samples from the abdominal aorta (Zeng et al., 2023) were aligned and counted, with output results being coherent with authors' calculations. To account for different depth coverage of RNA‐Seq, rarefication was performed, to equalize depth coverage between all samples, to mitigate noise introduced by data collected from two different sources (Bhide et al., 2014). Within DESeq2, genes with low expression (less than 10 reads across each sample) were removed based on raw read counts. The remaining raw read counts were then used to estimate size factors for sample normalization. Normalized read counts were used to fit the negative binomial model, compute mean and variance, and test the null hypothesis of equal gene expression in thoracic and abdominal conditions using a negative binomial test. Contrasting thoracic against abdominal samples, fold‐change differences, and *p*‐values were computed using the likelihood ratio test (LRT). Benjamini–Hochberg method adjusted *p*‐values and false discovery rates (FDR) were computed to control type I error. After all filtering amount of differentially expressed genes between the thoracic and the abdominal aorta was 497.

**FIGURE 2:**
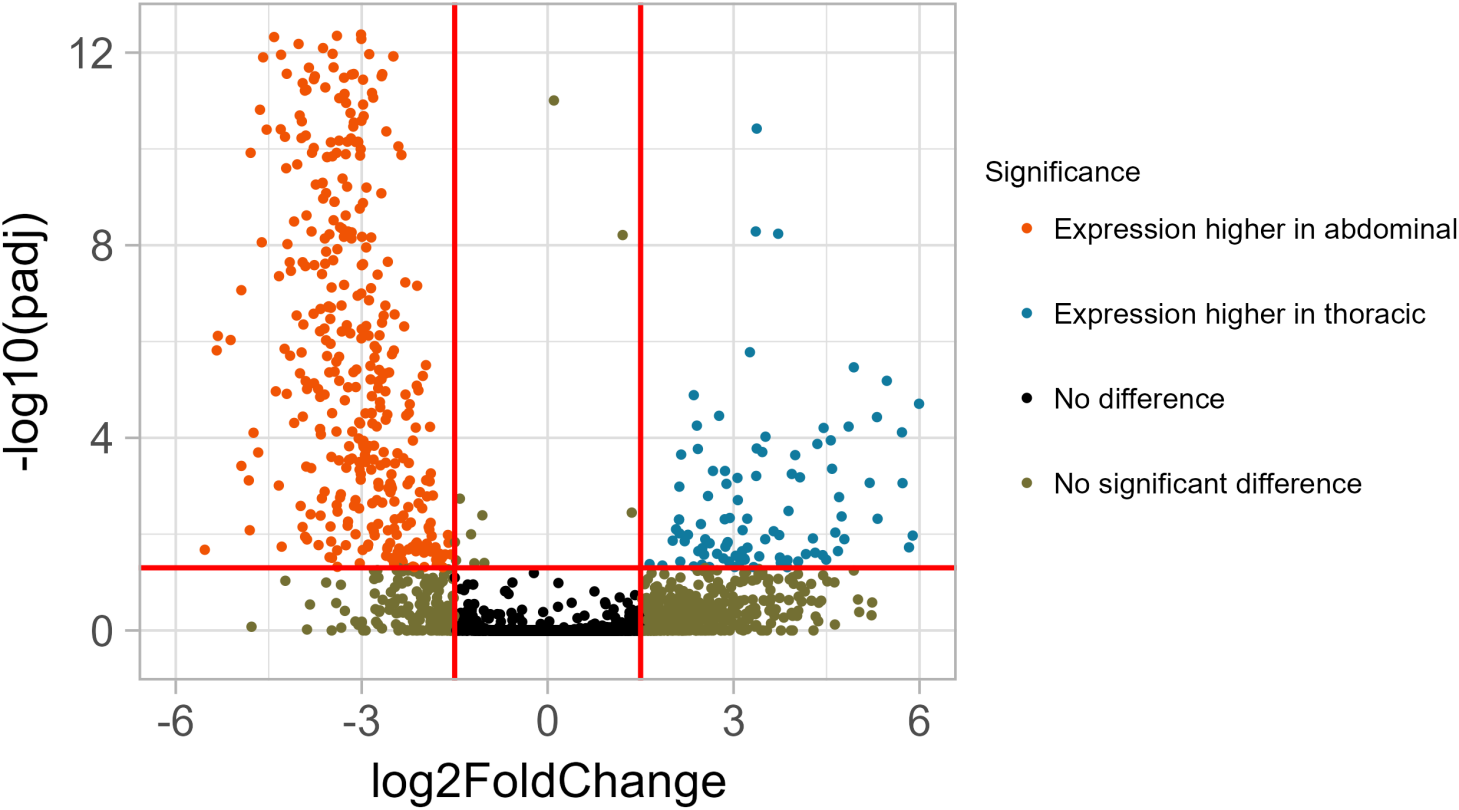
Thoracic aorta expression profile in comparison with the abdominal aorta. The volcano plot illustrates the log fold change (LFC) for the thoracic part of the aorta against the abdominal part. Orange dots signify underexpressed genes, while blue dots denote overexpressed genes. Additionally, brown and red dots represent genes with an LFC smaller than 1.5 and a *p*‐value higher than 0.05. This distinctive pattern indicates a discernible biological signal between aorta locations, highlighting the presence of differential expression between the two locations.

Differentially expressed genes were filtered based on criteria: Genes with <10 normalized read counts in each replicate were excluded, and only genes with FDR ≤0.05 and >1 fold difference between thoracic and abdominal conditions were retained. To address the higher variance in abdominal replicates, one abdominal sample was removed. To visually represent the outcomes of the analysis, a volcano plot was employed, revealing significant biological signals indicative of distinct expression patterns between the two aorta locations (Figure 2).

### 2.3 Statistical analysis of aorta relaxation data

The analysis began with the utilization of Clampfit software to obtain numerical values of tensile force in mN. The relaxation percentage was measured by the next formulae:

$$\text{Relaxation percentage}=\frac{\text{Relaxation for given case}}{\text{Basal tone}}\times 100.$$

Then, relaxation percentage for individual animals was averaged, to get one number for each animal. Following this, the analysis involved comparing the relaxation percentage values across different segments. When comparing two groups, the Welch two‐sample test was employed due to unequal variances and different sample sizes between groups (West, 2021). For comparisons involving more than two groups, Welch ANOVA was used (Delacre et al., 2019), with the Holm adjustment for multiple comparisons, with probabilities <5% were considered to be statistically significant (*p* < 0.05). All statistical analyses were carried out using R software.

To analyze results for basal tone, it was normalized according to the next formulae:

$$\text{Normalized basal tone}=\frac{\text{Basal tone}\;\mathrm{at}\;\text{given moment}}{\text{Initial basal tone}}.$$

This calculation was used to analyze data regarding changes in the basal tone of the vessel.

## 3 RESULTS

**FIGURE 3:**
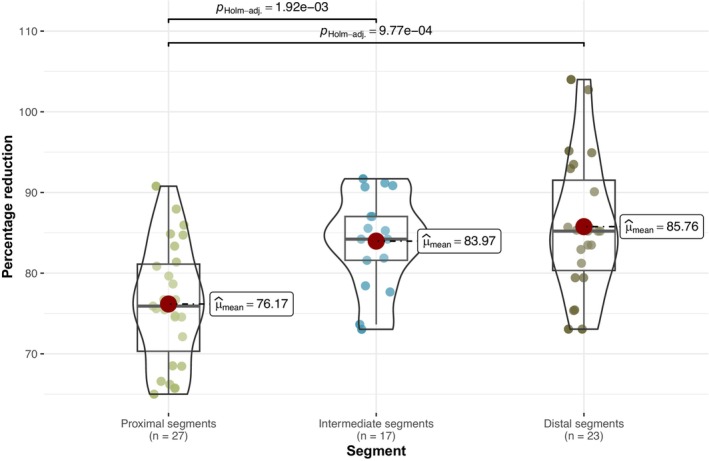
Comparison of acetylcholine‐induced relaxations on phenylephrine‐induced preconstriction between the thoracic aorta regions. In the comparison of acetylcholine‐induced relaxations on phenylephrine‐induced preconstriction between the thoracic aorta, proximal, intermediate, and distal segments were organized into pairs based on intercostal arteries and further grouped into adjusted pairs. A distinct statistical difference (*F*_(Welch)_ = 11.43, *p* = 1.14e^−4^, *n* = 67) is evident between the proximal, intermediate, and the distal segments.

To investigate the difference between proximal and distal thoracic aorta segments, percentage of acetylcholine‐induced relaxation on phenylephrine‐induced preconstriction were measured. Examination of acetylcholine‐induced relaxation amplitudes across aorta segments indicates statistically significant differences (*F*_(Welch)_ = 11.43, *p* = 1.14e^−4^, *n* = 67; Figure 3). While the precise reasons for these differences remain unclear, a hypothesis emerges: The uneven expression of certain ion channels, receptors, and structure proteins across the aorta wall may be a contributing factor.

To explore this hypothesis, we propose a reanalysis of openly available RNA‐seq data. This reexamination aims to identify genes that are likely linked to the observed differences in acetylcholine‐induced relaxation amplitudes, shedding light on potential molecular mechanisms underlying the functional variations along the length of the aorta.

### 3.1 RNA‐seq

Looking at the results of our differential expression, we see a significant biological signal between the thoracic and abdominal aorta. The filtered list of all differentially expressed genes which we found to be statistically significant can be found in the supplementary materials (Table S1).

Among the differentially expressed genes, several candidates emerge as likely targets that could account for the observed differences in acetylcholine‐induced relaxation amplitude across various segments of the aorta. To select the most promising candidates, we manually went through the list of all differentially expressed genes and selected those which are known from literature to be connected to endothelium‐dependent relaxation, or to vascular smooth muscle contraction—mainly ion channels, and components of muscular complex. These candidates include *Kcnj14*, *Cacna1*, *TUBA1*, *Arpc1b*, *Myl6*, and *Myo7b*.

*Kcnj14*, an inward rectifying potassium channel associated with EDHF‐induced relaxation, exhibits higher expression in the abdominal aorta compared to the thoracic region (LFC = −2.608389, padj = 5.829453e^−15^).

*Cacna1d*, a voltage gated calcium channel subunit (VGCC) alpha 1 D, also shows elevated expression in the abdominal aorta (LFC = −3.010768, padj = 4.212383e^−13^). This suggests a potential role in influencing the contractile behavior of the aorta segments.

*Tuba1*, a tubulin subunit alpha directly connected to gap junction functionalities, displays higher expression in the abdominal aorta. This heightened expression may contribute to an increased density of gap junctions in the abdominal region (LFC = −3.581549, padj = 1.476636e^−10^).

Finally, the higher expression of *Myl6* (LFC = −3.014067, padj = 5.661287e^−04^), a myosin light chain, *Myo7b*—myosin VIIb (LFC = −3.966135, padj = 2.669698e^−11^), *Arpc1b* (LFC = −2.725473, padj = 0.00574) an actin‐related protein, in the abdominal part of the aorta suggests a potential explanation for the observed differences in acetylcholine‐induced relaxation amplitudes.

### 3.2 Endothelium‐dependent relaxation in proximal and distal segments of the thoracic aorta locations

Upon analyzing our RNA‐seq results, we formulated a hypothesis suggesting that the differential expression of *Kcnj14* (K_IR_) and *Cacna1* (VGCC) genes could elucidate variations in amplitudes. To validate this hypothesis, we conducted a series of experiments measuring acetylcholine‐induced aorta relaxation on phenylephrine‐induced preconstriction while specifically targeting the blockade of K_IR_ and VGCC channels.

**FIGURE 4:**
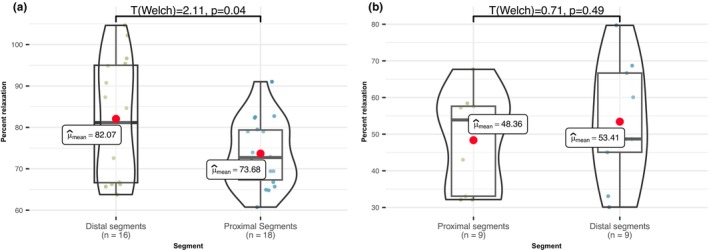
Comparison of acetylcholine‐induced relaxation amplitudes, in control and after potassium inward rectifying potassium blockade. (a) Noteworthy differences in acetylcholine‐induced relaxation percentages emerge between the proximal segments and distal segments. A significant distinction is evident (*t*_(welch)_ = 2.11, *p* = 0.04, *n* = 34), with distal segments displaying higher values. (b) Following the application of 100 μmol of Ba^2+^ and 40 μmol of ouabain, the difference in percentage of acetylcholine‐induced relaxations between the proximal segments and the distal segments dissipates (*t*_(welch)_ = −0.71, *p* = 0.49, *n* = 18), indicating that inhibition of K_IR_ channels inhibits relaxation in aorta strips, with more significant impact in the distal part of the thoracic aorta illuminating greater investment of K_IR_ channels in the regulation of abdominal part of the aorta.

In inhibiting the activity of the K_IR_ channels present on the SMC membrane, we employed Ba^2+^ and ouabain (100 and 40 μmol, respectively) to fully block potassium currents through K_IR_. Our findings indicate a notable outcome: following the pre‐application of Ba^2+^+Ouabain for 20 min, the previously observed sub‐significant differences in acetylcholine‐induced relaxation percentage between aortic segments disappeared (*t*_(welch)_ = −0.71, *p* = 0.49). In contrast, control measurements without receptor blockade maintained these differences (*t*_(welch)_ = 2.11, *p* = 0.04; Figure 4). This outcome partially supports our hypothesis, suggesting that the differential expression of K_IR_ indeed plays an important role in the observed phenomena of uneven acetylcholine‐induced relaxation percentage, through their impact on EDHF‐dependent relaxation.

This suggests that the density variance in K_IR_ channels might contribute to the observed differences in acetylcholine‐induced relaxations on phenylephrine‐induced preconstriction.

To investigate whether the variance in gap junction density, influenced by the differential expression of tubulin, could account for the observed distinctions in acetylcholine‐induced relaxation amplitudes, we conducted an experiment, employing 18GA (for 30 min) as a gap junction blocker. This should mitigate the effect of EDHF flowing through gap junctions, leaving only the transmembrane potassium component of EDHF active.

**FIGURE 5:**
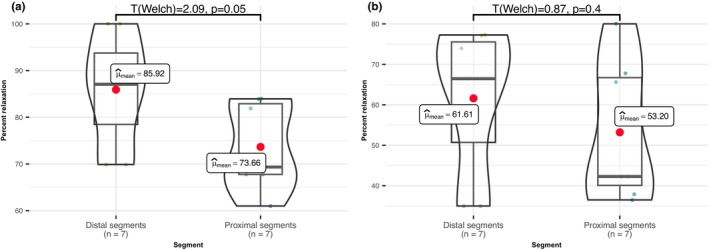
Difference in acetylcholine‐induced relaxation before and after 18α‐Glycyrrhetinic acid, in proximal and distal segments. (a) The disparity in acetylcholine‐induced relaxations between proximal segments and distal segments is evident, with statistically sub‐significant differences observed (*t*_(welch)_ = 2.09, *p* = 0.05, *n* = 14). Notably, the distal segments exhibit higher values. (b) Post the application of 10 μmol of 18GA, the difference in acetylcholine‐induced relaxations between the proximal segments and the distal segments diminishes (*t*_(welch)_ = 0.87, *p* = 0.4, *n* = 14), with more significant relaxation inhibition in distal segments. This phenomenon may be connected to a major role of gap junctions in EDHF propagation.

Our results revealed that post‐application of 18GA, the sub‐significant difference in acetylcholine‐induced relaxation percentage between proximal thoracic and distal thoracic segments disappeared (Figure 5; *t*_(welch)_ = 0.87, *p* = 0.4, *n* = 14), while in control recordings sub‐significant difference in relaxation percentage was still observed (*t*_(welch)_ = 2.09, *p* = 0.05, *n* = 14). This suggests that gap junctions, modulated by tubulin expression, possibly play a role in explaining the observed disparities in aortic behavior.

This suggests that the variance in gap junction density also plays a contributory role in the observed differences in acetylcholine‐induced relaxation, via gap junction connections to EDHF propagation.

In our final investigation, we sought to examine the potential correlation between the expression of *Cacna1* and variations in acetylcholine‐induced relaxation. To explore this, we employed nicardipine, a selective blocker of VGCC, to globally inhibit these channels.

**FIGURE 6:**
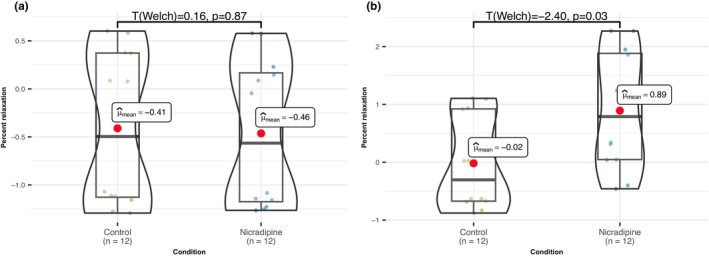
Role of voltage gated calcium channels on aorta rings basal tone. Comparison of normalized basal tone before and after nicardipine application in proximal (a) and distal (b) segments reveals a statistically significant difference only in the distal segments (*t*_(welch)_ = −2.40, *p* = 0.03, *n* = 24), while in proximal segments we don't see any significant difference (*t*_(welch)_ = 0.16, *p* = 0.87, *n* = 24) between control and post‐nicardipine basal tone.

Despite observing no noteworthy distinctions in the percentage of acetylcholine‐induced relaxation, additional analysis showed that in each experimental instance following the administration of nicardipine for 20 min, there was a discernible reduction in the basal tonus of distal segments (*t*_(welch)_ = −2.40, *p* = 0.03, *n* = 24; Figure 6). Concurrently, the proximal segments exhibited no significant decline in vascular tone (*t*_(welch)_ = 0.16, *p* = 0.87, *n* = 24). Consequently, it became evident that the dissimilar expression of *Cacna1* does not account for the observed differences in acetylcholine‐induced relaxation. Rather, its divergent expression appears to contribute to disparate behavior in proximal and distal thoracic aorta regions by basal tone regulation, due to a higher number of SMC in the downstream aorta part. This suggests that VGCC potentially plays a more crucial role in distal segments compared to their influence in proximal segments.

## 4 DISCUSSION

Our findings reveal a differential expression gradient between the abdominal and the thoracic aorta, providing a compelling explanation for observed variations in aortic behavior within the thoracic region. Despite the existence of a strict anatomical boundary, our study underscores the absence of a functional boundary between aorta regions, unveiling a functional gradient between the proximal and distal parts of the rat thoracic aorta. This intriguing discrepancy in expression may contribute to the observed differences in acetylcholine‐induced relaxation on phenylephrine preconstriction amplitudes between thoracic aortic segments due to K_IR_ well‐known role in aorta relaxation implicating the involvement of EDHF and NO (Ahn et al., 2017; Honda et al., 1997; Jackson, 2017), while gap junctions are impacting the propagation of EDHF from endothelium to smooth muscle cells, and therefore to vessel relaxation (Giepmans et al., 2001; Schmidt & de Wit, 2020).

Outside of the potassium inward rectifying channel, another potassium channel—*Kcnk3*, showed sub‐significant (LFC = 4.03893458, padj = 0.067) differential expression, with higher expression in the proximal part. This channel belongs to the two pore domain potassium channels subfamily, which is known to play an important role in regulating resting membrane potential through K^+^ leak (Makino et al., 2011). Our data cannot provide distinctive results on this channel, differential expression, yet it may be important to explain differences in proximal and distal aorta locations.

Another interesting pattern is connected to the differential expression of VGCC, linked to cellular excitation and calcium signaling crucial for muscular contractility (Fan et al., 2019; Braunstein et al., 2009; Moosmang et al., 2003), which we show to play a role in basal tone regulation for proximal thoracic aorta segments, and may be additionally regulated by K_IR_, with VGCC being deactivated by hyperpolarization, and therefore causing lesser constriction, what shows possible intercorrelation between higher expression of K_IR_ and VGCC.

Also, results of our differential expression analysis showed higher expression of muscular complex components (Myl6, Myo7b, Arpc1b), in the distal thoracic aorta region. This may indicate a higher density of muscular fibers in the distal regions of the aorta, influencing its contractile behavior, which aligns with previous research about aorta anatomy (Sanders, 2008; Shahoud et al., 2023).

Our results align seamlessly with existing literature on aorta anatomy, where the proximal parts of the thoracic aorta exhibit elastic functions, acting as buffers against blood pressure spikes, as confirmed by lower myosinization and smaller density of elements responsible for relaxation. In contrast, the distal regions play a pivotal role in regulating the supply to thoracic cavity organs through their contractile properties, as evidenced by higher levels of myosin expression and a higher density of contraction and relaxation‐regulating elements.

Furthermore, our in‐depth RNA‐SEQ analysis goes beyond confirming the expression gradient connected to relaxation by identifying additional differentially expressed genes associated with other physiological functions. These findings offer valuable insights that extend our understanding of the spatial patterns governing aortic behavior. The identified genes may play crucial roles in shaping the intricate dynamics of aortic function across different regions, contributing significantly to our comprehension of vascular dynamics, while other mechanism related to disparities in aorta reactions between different regions is to be unveiled.

### 4.1 Perspectives and significance

In summary, our study has demonstrated functional differences between the proximal and distal regions of the thoracic aorta. Utilizing RNA‐Seq analysis, we revealed distinct differential expression patterns between the thoracic and abdominal aortas. Subsequently, experimental validation confirmed the functional disparities related to K_IR_, gap junction, VGCC, and, consequently, EDHF in the proximal and distal regions of the thoracic aorta, highlighting the functional heterogeneity within the aorta. These conclusions are crucial for understanding rat vascular dynamics and the regulatory mechanisms governing blood supply to its organs.

### 4.2 Study limitations

While RNA‐Seq data analysis offers valuable insights into understanding differential expression profiles across experimental conditions, it is susceptible to noise arising from various factors such as different batches. Given the nature of our analysis, where we utilize sequencing data from two different articles, it was challenging to implement batch correction and mitigate noise introduced by gathering data from distinct sources.

**FIGURE 7:**
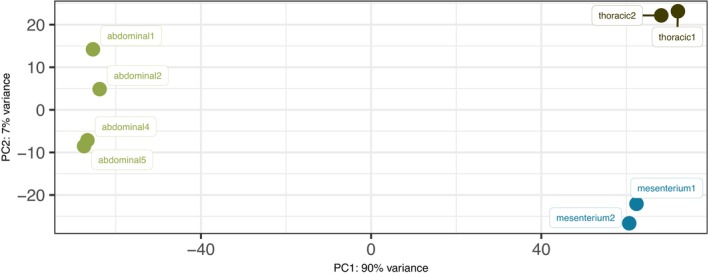
PCA plot showing the distance between thoracic, abdominal, and mesenterial tissues samples.

In an effort to validate our analysis, we examined the variance between two aorta locations and other rat tissues. To assess this, we employed principal component analysis (PCA) and plotted the results. Our analysis indicates that the difference between all tissues tends to be significant, and all tissue‐specific samples can be grouped together (Figure 7). This partially confirms the validity of our analysis.

Furthermore, the results of our RNA‐Seq data are partially corroborated by our relaxation measurements, providing evidence of the differential expression of *Kcnj14*, *Cacna1d*, and *Tuba1* genes. This additional validation adds confidence to the reliability of our findings.

## AUTHOR CONTRIBUTIONS

O.R. developed the hypothesis, performed the data analysis, performed animal experiments, and analyzed RNA data. Both O.R and Ph.I. authors contributed to the final version of the manuscript. Ph.I. supervised the project.

## FUNDING INFORMATION

The study was carried out with the assistance of the Ukraine National Research Foundation grants 2020/02/0189.

## CONFLICT OF INTEREST STATEMENT

There is no conflict of interest.

## DISCLAIMERS

Such as for Government agency work.

## Supporting information


Table S1.

## Data Availability

All data and corresponding analysis notebooks are available at the corresponding git. Data regarding RNA‐Seq belong to responsible authors and can be found in the Gene Expression Omnibus database by IDs—GSE64450 and GSE214655.
