# Supplementary material for: The difference in endothelium‐dependent relaxation components in proximal and distal thoracic aorta regions of male rats
Source: Physiol Rep. 2024 Mar 27;12(6):e15992. doi: 10.14814/phy2.15992 (PMC10972677; doi:10.14814/phy2.15992)
Supplement: Supplementary file 1 — Table S1. [file PHY2-12-e15992-s001.docx]

| Gene Transcript ID | Base mean counts | Log2FoldChange | Log2FoldChange standard error | svalue | Adjusted pvalue | Gene_biotype | External gene name | Description |
| --- | --- | --- | --- | --- | --- | --- | --- | --- |
| ENSRNOT00000017454 | 39,61701 | 7,770678 | 1,413592 | 6,81E-10 | 0,000881 | protein_coding | Aspg | asparaginase [Source:RGD Symbol;Acc:708388] |
| ENSRNOT00000013238 | 41,76505 | 6,90987 | 1,026102 | 2,19E-13 | 0,000103 | protein_coding | Acadm | acyl-CoA dehydrogenase medium chain [Source:RGD Symbol;Acc:2012] |
| ENSRNOT00000067441 | 37,39796 | 6,82589 | 1,284937 | 2,01E-09 | 0,000746 | protein_coding | Rps15-ps2 | ribosomal protein S15, pseudogene 2 [Source:RGD Symbol;Acc:1587266] |
| ENSRNOT00000115047 | 45,75626 | 6,496665 | 0,895981 | 5,36E-15 | 0,000754 | protein_coding | Gnas | GNAS complex locus [Source:RGD Symbol;Acc:2716] |
| ENSRNOT00000002310 | 64,91238 | 6,362989 | 1,32694 | 3,53E-08 | 0,001584 | protein_coding | Cbr3 | carbonyl reductase 3 [Source:RGD Symbol;Acc:1309728] |
| ENSRNOT00000068694 | 39,21553 | 6,114006 | 1,000599 | 1,71E-11 | 0,002447 | protein_coding | AABR07001902.1 | |
| ENSRNOT00000026711 | 78,91417 | 6,077164 | 0,696954 | 3,81E-20 | 0,000426 | protein_coding | Mst1 | macrophage stimulating 1 [Source:RGD Symbol;Acc:3114] |
| ENSRNOT00000074950 | 46,02086 | 5,992211 | 0,759046 | 3,34E-17 | 1,96E-05 | protein_coding | Ndufa3-ps3 | NADH:ubiquinone oxidoreductase subunit A3, pseudogene 3 [Source:RGD Symbol;Acc:1589176] |
| ENSRNOT00000020182 | 37,79764 | 5,725934 | 1,282881 | 1,71E-07 | 0,000872 | protein_coding | Sfrp5 | secreted frizzled-related protein 5 [Source:RGD Symbol;Acc:1310369] |
| ENSRNOT00000104742 | 74,31434 | 5,71634 | 0,617339 | 1,88E-22 | 7,69E-05 | protein_coding | Atp5mf | ATP synthase membrane subunit f [Source:RGD Symbol;Acc:1596067] |
| ENSRNOT00000072464 | 88,54581 | 5,472518 | 0,570314 | 7,37E-24 | 6,54E-06 | protein_coding | Romo1 | reactive oxygen species modulator 1 [Source:RGD Symbol;Acc:1587111] |
| ENSRNOT00000046201 | 6409,827 | 5,32324 | 0,508473 | 1,04E-27 | 0,004795 | protein_coding | Mt-atp8 | mitochondrially encoded ATP synthase membrane subunit 8 [Source:RGD Symbol;Acc:621240] |
| ENSRNOT00000009681 | 93,97657 | 5,313284 | 0,52418 | 3,75E-26 | 3,71E-05 | protein_coding | Idh3b | isocitrate dehydrogenase (NAD(+)) 3 non-catalytic subunit beta [Source:RGD Symbol;Acc:621881] |
| ENSRNOT00000073930 | 92,2838 | 5,195722 | 0,606111 | 1,12E-19 | 0,000861 | protein_coding | Plin5 | perilipin 5 [Source:RGD Symbol;Acc:1589602] |
| ENSRNOT00000101188 | 156,4479 | 4,939777 | 0,432546 | 3,92E-32 | 3,43E-06 | protein_coding | Cox5a | cytochrome c oxidase subunit 5A [Source:RGD Symbol;Acc:620607] |
| ENSRNOT00000033253 | 224,7832 | 4,855918 | 0,437185 | 1,25E-30 | 5,84E-05 | protein_coding | LOC100361008 | Cytochrome c oxidase subunit 5A, mitochondrial-like [Source:RGD Symbol;Acc:2320952] |
| ENSRNOT00000110622 | 41,22277 | 4,742928 | 0,700967 | 1,99E-13 | 0,004288 | protein_coding | H3f3a-ps9 | H3.3 histone A, pseudogene 9 [Source:RGD Symbol;Acc:2322285] |
| ENSRNOT00000026408 | 690,834 | 4,701875 | 0,451811 | 2,8E-27 | 0,001701 | protein_coding | Agpat2 | 1-acylglycerol-3-phosphate O-acyltransferase 2 [Source:RGD Symbol;Acc:1309229] |
| ENSRNOT00000031472 | 487,7317 | 4,641103 | 0,502507 | 2,64E-22 | 0,009279 | protein_coding | Chchd10 | coiled-coil-helix-coiled-coil-helix domain containing 10 [Source:RGD Symbol;Acc:1359417] |
| ENSRNOT00000071913 | 305,4189 | 4,588866 | 0,42128 | 1,32E-29 | 0,000439 | protein_coding | Atp5f1e | ATP synthase F1 subunit epsilon [Source:RGD Symbol;Acc:621374] |
| ENSRNOT00000001625 | 212,0487 | 4,568172 | 0,412523 | 1,95E-30 | 0,000113 | protein_coding | Pfkl | phosphofructokinase, liver type [Source:RGD Symbol;Acc:3311] |
| ENSRNOT00000044582 | 5701,056 | 4,452827 | 0,381233 | 1,99E-33 | 6,23E-05 | protein_coding | Mt-nd4l | mitochondrially encoded NADH:ubiquinone oxidoreductase core subunit 4L [Source:RGD Symbol;Acc:620494] |
| ENSRNOT00000004602 | 192,3922 | 4,35204 | 0,396232 | 5,51E-30 | 0,000134 | protein_coding | Adora1 | adenosine A1 receptor [Source:RGD Symbol;Acc:2048] |
| ENSRNOT00000110214 | 128,3564 | 4,071477 | 0,479548 | 2,35E-19 | 0,000661 | protein_coding | Cidea | cell death-inducing DFFA-like effector a [Source:RGD Symbol;Acc:1305106] |
| ENSRNOT00000075493 | 116,5513 | 3,994166 | 0,382797 | 1,7E-27 | 0,000228 | protein_coding | Fabp5 | fatty acid binding protein 5 [Source:RGD Symbol;Acc:70997] |
| ENSRNOT00000000072 | 231,5694 | 3,939141 | 0,370472 | 2,33E-28 | 0,000563 | protein_coding | Atp5me | ATP synthase membrane subunit e [Source:RGD Symbol;Acc:621377] |
| ENSRNOT00000100770 | 413,9261 | 3,886427 | 0,385121 | 6,58E-26 | 0,003295 | protein_coding | Uqcrq | ubiquinol-cytochrome c reductase, complex III subunit VII [Source:RGD Symbol;Acc:1562350] |
| ENSRNOT00000083839 | 413,6521 | 3,71946 | 0,280138 | 3,48E-42 | 5,78E-09 | protein_coding | Uqcr10 | ubiquinol-cytochrome c reductase, complex III subunit X [Source:RGD Symbol;Acc:1595126] |
| ENSRNOT00000045705 | 87,79333 | 3,646848 | 0,401335 | 1,07E-21 | 0,00869 | protein_coding | LOC100361934 | NADH dehydrogenase (ubiquinone) 1 beta subcomplex 4 [Source:RGD Symbol;Acc:2319146] |
| ENSRNOT00000054965 | 201,457 | 3,514933 | 0,320463 | 6,36E-30 | 9,47E-05 | protein_coding | Mrpl12 | mitochondrial ribosomal protein L12 [Source:RGD Symbol;Acc:1588559] |
| ENSRNOT00000022892 | 185,1054 | 3,463555 | 0,319702 | 3,27E-29 | 0,000197 | protein_coding | Atp5f1a | ATP synthase F1 subunit alpha [Source:RGD Symbol;Acc:619993] |
| ENSRNOT00000066670 | 559,1846 | 3,372941 | 0,239119 | 8,66E-47 | 3,78E-11 | protein_coding | Cxcl12 | C-X-C motif chemokine ligand 12 [Source:RGD Symbol;Acc:3651] |
| ENSRNOT00000094138 | 168,2582 | 3,372718 | 0,344658 | 1,4E-24 | 0,000166 | protein_coding | RGD1564963 | similar to ribosomal protein L10 [Source:RGD Symbol;Acc:1564963] |
| ENSRNOT00000083629 | 112,6092 | 3,364948 | 0,33015 | 1,62E-26 | 0,000619 | protein_coding | Akr7a2 | aldo-keto reductase family 7, member A2 [Source:RGD Symbol;Acc:620311] |
| ENSRNOT00000004583 | 286,4306 | 3,357655 | 0,271508 | 5,9E-37 | 5,17E-09 | protein_coding | AABR07029195.1 | |
| ENSRNOT00000057895 | 340,4401 | 3,263015 | 0,296618 | 4,15E-30 | 1,67E-06 | protein_coding | Rpl34-ps1 | ribosomal protein L34, pseudogene 1 [Source:RGD Symbol;Acc:1595454] |
| ENSRNOT00000022487 | 489,9832 | 3,220084 | 0,336954 | 1,76E-23 | 0,00478 | protein_coding | Cox5b | cytochrome c oxidase subunit 5B [Source:RGD Symbol;Acc:620608] |
| ENSRNOT00000003557 | 147,5901 | 3,145626 | 0,343237 | 4,81E-22 | 0,008263 | protein_coding | LOC100361993 | transmembrane protein 14C [Source:RGD Symbol;Acc:2319087] |
| ENSRNOT00000027615 | 221,1301 | 3,068793 | 0,337126 | 9,62E-22 | 0,001963 | protein_coding | Slc25a42 | solute carrier family 25, member 42 [Source:RGD Symbol;Acc:1592346] |
| ENSRNOT00000022277 | 51,35376 | 3,062413 | 0,372901 | 2,25E-18 | 0,00068 | protein_coding | Pllp | plasmolipin [Source:RGD Symbol;Acc:621478] |
| ENSRNOT00000016051 | 219,0125 | 2,938252 | 0,306313 | 8,39E-24 | 0,004613 | protein_coding | Ndufb5 | NADH:ubiquinone oxidoreductase subunit B5 [Source:RGD Symbol;Acc:1305909] |
| ENSRNOT00000017325 | 163,6994 | 2,88509 | 0,383977 | 7,88E-16 | 0,000903 | protein_coding | Fabp3 | fatty acid binding protein 3 [Source:RGD Symbol;Acc:69048] |
| ENSRNOT00000019797 | 326,2143 | 2,86194 | 0,272618 | 7,44E-28 | 0,000487 | protein_coding | Pdha1l1 | pyruvate dehydrogenase (lipoamide) alpha 1-like 1 [Source:RGD Symbol;Acc:1590190] |
| ENSRNOT00000075424 | 1153,44 | 2,857157 | 0,306343 | 1,31E-22 | 0,004908 | protein_coding | Plin4 | perilipin 4 [Source:RGD Symbol;Acc:1590867] |
| ENSRNOT00000010556 | 241,5366 | 2,766195 | 0,243052 | 7,72E-32 | 3,48E-05 | protein_coding | Adipor2 | adiponectin receptor 2 [Source:RGD Symbol;Acc:1307891] |
| ENSRNOT00000019923 | 165,8722 | 2,667432 | 0,261749 | 1,91E-26 | 0,000487 | protein_coding | Mlycd | malonyl-CoA decarboxylase [Source:RGD Symbol;Acc:620234] |
| ENSRNOT00000001602 | 287,3425 | 2,587224 | 0,255908 | 5,01E-26 | 0,001614 | protein_coding | Agpat3 | 1-acylglycerol-3-phosphate O-acyltransferase 3 [Source:RGD Symbol;Acc:1305787] |
| ENSRNOT00000032787 | 442,0275 | 2,472795 | 0,264988 | 1,19E-22 | 0,006178 | protein_coding | Rpsa-ps4 | ribosomal protein SA, pseudogene 4 [Source:RGD Symbol;Acc:1588760] |
| ENSRNOT00000001609 | 406,7487 | 2,423604 | 0,223027 | 2,01E-29 | 0,00017 | protein_coding | Gatd3a | glutamine amidotransferase class 1 domain containing 3A [Source:RGD Symbol;Acc:1303003] |
| ENSRNOT00000049520 | 290,8636 | 2,407551 | 0,230559 | 1,46E-27 | 5,58E-05 | protein_coding | Gm5471 | predicted pseudogene 5471 [Source:RGD Symbol;Acc:1560994] |
| ENSRNOT00000025794 | 813,373 | 2,357766 | 0,205951 | 3,33E-32 | 1,3E-05 | protein_coding | Sod2 | superoxide dismutase 2 [Source:RGD Symbol;Acc:3732] |
| ENSRNOT00000022022 | 244,6856 | 2,153906 | 0,226294 | 2,65E-23 | 0,000222 | protein_coding | Eci2 | enoyl-CoA delta isomerase 2 [Source:RGD Symbol;Acc:1359427] |
| ENSRNOT00000008200 | 279,3871 | 2,126658 | 0,245165 | 4,9E-20 | 0,009624 | protein_coding | Lrpprc | leucine-rich pentatricopeptide repeat containing [Source:RGD Symbol;Acc:1306575] |
| ENSRNOT00000097334 | 153,708 | 2,125136 | 0,286237 | 1,56E-15 | 0,001033 | protein_coding | Ccdc85b | coiled-coil domain containing 85B [Source:RGD Symbol;Acc:1565319] |
| ENSRNOT00000046519 | 225,0798 | 2,120607 | 0,236062 | 2,91E-21 | 0,004981 | protein_coding | Rpl37l1 | ribosomal protein L37-like1 [Source:RGD Symbol;Acc:621204] |
| ENSRNOT00000008262 | 75,59344 | 2,071692 | 0,621905 | 2,57E-05 | 0,007928 | protein_coding | Spon2 | spondin 2 [Source:RGD Symbol;Acc:708584] |
| ENSRNOT00000032387 | 744,1535 | -1,85144 | 0,205932 | 2,63E-21 | 0,001576 | protein_coding | Scyl1 | SCY1 like pseudokinase 1 [Source:RGD Symbol;Acc:1307330] |
| ENSRNOT00000036634 | 410,7715 | -1,88477 | 0,235603 | 1,42E-17 | 0,000549 | protein_coding | Pdf | peptide deformylase (mitochondrial) [Source:RGD Symbol;Acc:1582894] |
| ENSRNOT00000037332 | 505,5743 | -1,88768 | 0,226767 | 8,35E-19 | 0,005818 | protein_coding | Dnajc14 | DnaJ heat shock protein family (Hsp40) member C14 [Source:RGD Symbol;Acc:620489] |
| ENSRNOT00000068747 | 267,5312 | -1,90324 | 0,286805 | 4,98E-13 | 0,000801 | protein_coding | Rsph9 | radial spoke head component 9 [Source:RGD Symbol;Acc:1310693] |
| ENSRNOT00000044292 | 316,0946 | -1,90815 | 0,235155 | 6,2E-18 | 0,008093 | protein_coding | Ormdl2 | ORMDL sphingolipid biosynthesis regulator 2 [Source:RGD Symbol;Acc:1305642] |
| ENSRNOT00000108773 | 280,8184 | -1,91965 | 0,293999 | 1,11E-12 | 0,007139 | protein_coding | Ppp2r5d | protein phosphatase 2, regulatory subunit B', delta [Source:RGD Symbol;Acc:1306666] |
| ENSRNOT00000106436 | 419,2626 | -1,94721 | 0,264958 | 3,13E-15 | 0,001645 | protein_coding | RGD1563578 | similar to PGC7 [Source:RGD Symbol;Acc:1563578] |
| ENSRNOT00000071978 | 105,3867 | -1,9597 | 0,264598 | 1,75E-15 | 0,000759 | protein_coding | Pdss1 | decaprenyl diphosphate synthase subunit 1 [Source:RGD Symbol;Acc:2319976] |
| ENSRNOT00000015562 | 314,5311 | -1,96342 | 0,195369 | 1,06E-25 | 3,09E-06 | protein_coding | Usp39 | ubiquitin specific peptidase 39 [Source:RGD Symbol;Acc:1308103] |
| ENSRNOT00000075236 | 2002,27 | -2,01385 | 0,228915 | 1,63E-20 | 5,17E-06 | protein_coding | Cox7a1 | cytochrome c oxidase subunit 7A1 [Source:RGD Symbol;Acc:6496527] |
| ENSRNOT00000096877 | 448,8807 | -2,0235 | 0,286271 | 2,27E-14 | 0,002898 | protein_coding | Abhd8 | abhydrolase domain containing 8 [Source:RGD Symbol;Acc:1305693] |
| ENSRNOT00000107276 | 93,354 | -2,06098 | 0,303274 | 1,58E-13 | 0,001341 | protein_coding | Ccnc | cyclin C [Source:RGD Symbol;Acc:70905] |
| ENSRNOT00000041645 | 934,4465 | -2,08316 | 0,270828 | 1,24E-16 | 0,002299 | protein_coding | Gusb | glucuronidase, beta [Source:RGD Symbol;Acc:2772] |
| ENSRNOT00000003284 | 802,0993 | -2,08438 | 0,2233 | 1,08E-22 | 1,03E-05 | protein_coding | Pdxdc1 | pyridoxal-dependent decarboxylase domain containing 1 [Source:RGD Symbol;Acc:1562597] |
| ENSRNOT00000013542 | 376,8589 | -2,10559 | 0,225313 | 8,52E-23 | 6,95E-08 | protein_coding | Lrrc46 | leucine rich repeat containing 46 [Source:RGD Symbol;Acc:1303311] |
| ENSRNOT00000101020 | 397,05 | -2,11194 | 0,208281 | 3,25E-26 | 8,25E-06 | protein_coding | LOC100912483 | uncharacterized LOC100912483 [Source:RGD Symbol;Acc:6486589] |
| ENSRNOT00000079988 | 1370,318 | -2,1277 | 0,214872 | 3,52E-25 | 6,13E-05 | protein_coding | AC115420.4 | |
| ENSRNOT00000105468 | 164,1503 | -2,17402 | 0,258828 | 5,62E-19 | 0,000114 | protein_coding | Aamdc | adipogenesis associated, Mth938 domain containing [Source:RGD Symbol;Acc:1561459] |
| **ENSRNOT00000114214** | **128,2232** | **-2,20823** | **0,272924** | **7,66E-18** | **0,005745** | **protein_coding** | **Arpc1b** | **actin related protein 2/3 complex, subunit 1B [Source:RGD Symbol;Acc:2155]** |
| ENSRNOT00000005078 | 339,3565 | -2,2235 | 0,256671 | 6,21E-20 | 0,000771 | protein_coding | Zdhhc17 | zinc finger DHHC-type palmitoyltransferase 17 [Source:RGD Symbol;Acc:1595790] |
| ENSRNOT00000014814 | 368,8874 | -2,22466 | 0,273382 | 5,77E-18 | 2E-05 | protein_coding | Fam161b | FAM161 centrosomal protein B [Source:RGD Symbol;Acc:1309058] |
| ENSRNOT00000116906 | 393,7238 | -2,23919 | 0,313515 | 1,12E-14 | 0,000333 | protein_coding | Wdr46 | WD repeat domain 46 [Source:RGD Symbol;Acc:2209] |
| ENSRNOT00000056531 | 386,1511 | -2,2395 | 0,291411 | 1,37E-16 | 3,04E-05 | protein_coding | Cep63 | centrosomal protein 63 [Source:RGD Symbol;Acc:1561183] |
| ENSRNOT00000083097 | 277,7125 | -2,24811 | 0,259816 | 7,6E-20 | 0,002737 | protein_coding | B3gnt9 | UDP-GlcNAc:betaGal beta-1,3-N-acetylglucosaminyltransferase 9 [Source:RGD Symbol;Acc:1310170] |
| ENSRNOT00000026789 | 1246,485 | -2,26025 | 0,236182 | 1,21E-23 | 0,000919 | protein_coding | Jmjd8 | jumonji domain containing 8 [Source:RGD Symbol;Acc:1307381] |
| ENSRNOT00000109008 | 192,0488 | -2,27289 | 0,328981 | 6,41E-14 | 0,006681 | protein_coding | Hspa12a | heat shock protein family A (Hsp70) member 12A [Source:RGD Symbol;Acc:1305531] |
| ENSRNOT00000090519 | 1313,576 | -2,28181 | 0,234789 | 2,19E-24 | 3,44E-05 | protein_coding | Arhgap35 | Rho GTPase activating protein 35 [Source:RGD Symbol;Acc:1308738] |
| ENSRNOT00000085988 | 213,6165 | -2,29349 | 0,238788 | 6,37E-24 | 1,26E-05 | protein_coding | Idh2 | isocitrate dehydrogenase (NADP(+)) 2 [Source:RGD Symbol;Acc:1597139] |
| ENSRNOT00000112738 | 161,2775 | -2,29666 | 0,234175 | 9,7E-25 | 5,92E-08 | protein_coding | Car14 | carbonic anhydrase 14 [Source:RGD Symbol;Acc:1599277] |
| ENSRNOT00000029274 | 549,9903 | -2,31479 | 0,293631 | 3,61E-17 | 4,85E-07 | protein_coding | Tmem241 | transmembrane protein 241 [Source:RGD Symbol;Acc:1590602] |
| ENSRNOT00000066007 | 127,4356 | -2,32665 | 0,318767 | 4,46E-15 | 0,004087 | protein_coding | Dnm2 | dynamin 2 [Source:RGD Symbol;Acc:2513] |
| ENSRNOT00000111275 | 453,1762 | -2,329 | 0,274955 | 2,8E-19 | 0,000265 | protein_coding | Cdc37l1 | cell division cycle 37-like 1 [Source:RGD Symbol;Acc:1309295] |
| ENSRNOT00000001829 | 507,92 | -2,35885 | 0,195058 | 1,27E-35 | 1,33E-10 | protein_coding | Taf6 | TATA-box binding protein associated factor 6 [Source:RGD Symbol;Acc:1311608] |
| ENSRNOT00000104637 | 135,3303 | -2,36804 | 0,290549 | 5,07E-18 | 0,00463 | protein_coding | LOC103691238 | zinc finger protein 239-like [Source:RGD Symbol;Acc:9074604] |
| ENSRNOT00000024440 | 482,1175 | -2,40809 | 0,192562 | 6,48E-38 | 8,85E-11 | protein_coding | Farp2 | FERM, ARH/RhoGEF and pleckstrin domain protein 2 [Source:RGD Symbol;Acc:1308003] |
| ENSRNOT00000103808 | 795,0433 | -2,41408 | 0,209603 | 1,22E-32 | 2,97E-13 | protein_coding | Pfdn2 | prefoldin subunit 2 [Source:RGD Symbol;Acc:1591406] |
| ENSRNOT00000067932 | 191,6802 | -2,42366 | 0,249958 | 3,25E-24 | 0,000209 | protein_coding | Alg1 | ALG1, chitobiosyldiphosphodolichol beta-mannosyltransferase [Source:RGD Symbol;Acc:1310700] |
| ENSRNOT00000010180 | 879,5327 | -2,46304 | 0,277869 | 7,56E-21 | 0,000339 | protein_coding | Cd63 | Cd63 molecule [Source:RGD Symbol;Acc:62080] |
| ENSRNOT00000019689 | 775,406 | -2,46885 | 0,224563 | 4,79E-30 | 2,73E-07 | protein_coding | Brf1 | BRF1, RNA polymerase III transcription initiation factor subunit [Source:RGD Symbol;Acc:1311158] |
| ENSRNOT00000043247 | 94,28809 | -2,4721 | 0,312677 | 2,86E-17 | 0,002098 | protein_coding | AABR07071482.1 | |
| ENSRNOT00000054913 | 87,09248 | -2,47601 | 0,32138 | 1,12E-16 | 0,007628 | protein_coding | Tbcd | tubulin folding cofactor D [Source:RGD Symbol;Acc:2320148] |
| ENSRNOT00000091000 | 702,2234 | -2,48091 | 0,259771 | 1,91E-23 | 1,53E-06 | protein_coding | Rcbtb2 | RCC1 and BTB domain containing protein 2 [Source:RGD Symbol;Acc:735048] |
| ENSRNOT00000101966 | 300,2879 | -2,48946 | 0,227437 | 9,48E-30 | 1,2E-12 | protein_coding | Ndufb9 | NADH:ubiquinone oxidoreductase subunit B9 [Source:RGD Symbol;Acc:1307114] |
| ENSRNOT00000102365 | 294,7078 | -2,51104 | 0,237135 | 3,96E-28 | 1,83E-06 | protein_coding | Trappc2 | trafficking protein particle complex subunit 2 [Source:RGD Symbol;Acc:1306925] |
| ENSRNOT00000114295 | 333,567 | -2,51265 | 0,306375 | 2,46E-18 | 0,001107 | protein_coding | Lrwd1 | leucine-rich repeats and WD repeat domain containing 1 [Source:RGD Symbol;Acc:1359442] |
| ENSRNOT00000096750 | 619,4646 | -2,51982 | 0,272498 | 2,36E-22 | 0,000571 | protein_coding | Sowahc | sosondowah ankyrin repeat domain family member C [Source:RGD Symbol;Acc:1595666] |
| ENSRNOT00000007407 | 109,9724 | -2,52665 | 0,329592 | 1,78E-16 | 0,000969 | protein_coding | LOC686013 | RIKEN cDNA 1700067K01 gene [Source:MGI Symbol;Acc:MGI:1920703] |
| ENSRNOT00000114659 | 146,5465 | -2,53715 | 0,292382 | 4,49E-20 | 0,001336 | protein_coding | B3gat3 | beta-1,3-glucuronyltransferase 3 [Source:RGD Symbol;Acc:1311968] |
| ENSRNOT00000026873 | 121,9225 | -2,53984 | 0,28194 | 1,91E-21 | 0,000884 | protein_coding | Esrp2 | epithelial splicing regulatory protein 2 [Source:RGD Symbol;Acc:1310855] |
| ENSRNOT00000119595 | 183,2368 | -2,55742 | 0,24781 | 4,5E-27 | 4,39E-06 | protein_coding | Crb3 | crumbs cell polarity complex component 3 [Source:RGD Symbol;Acc:1565035] |
| ENSRNOT00000057073 | 657,8937 | -2,5702 | 0,279056 | 3,33E-22 | 0,001108 | protein_coding | Lrfn1 | leucine rich repeat and fibronectin type III domain containing 1 [Source:RGD Symbol;Acc:1304707] |
| ENSRNOT00000092475 | 113,8732 | -2,57681 | 0,325364 | 2,43E-17 | 0,008322 | protein_coding | Srr | serine racemase [Source:RGD Symbol;Acc:735094] |
| ENSRNOT00000101611 | 506,7075 | -2,57848 | 0,230095 | 5,1E-31 | 2,21E-08 | protein_coding | Mtif3 | mitochondrial translational initiation factor 3 [Source:RGD Symbol;Acc:1592673] |
| ENSRNOT00000119067 | 133,3194 | -2,58641 | 0,282917 | 7,27E-22 | 3,29E-05 | protein_coding | Uty | ubiquitously transcribed tetratricopeptide repeat containing, Y-linked [Source:RGD Symbol;Acc:1565481] |
| ENSRNOT00000005170 | 1142,015 | -2,60225 | 0,202771 | 1,63E-39 | 4,33E-11 | protein_coding | Ppp4r3b | protein phosphatase 4, regulatory subunit 3B [Source:RGD Symbol;Acc:1309450] |
| **ENSRNOT00000028591** | **2417,478** | **-2,60839** | **0,167679** | **2,13E-56** | **5,83E-15** | **protein_coding** | **Kcnj14** | **potassium inwardly-rectifying channel, subfamily J, member 14 [Source:RGD Symbol;Acc:628872]** |
| ENSRNOT00000104624 | 560,4082 | -2,61241 | 0,26557 | 6,27E-25 | 4,2E-05 | protein_coding | Prdx5 | peroxiredoxin 5 [Source:RGD Symbol;Acc:71007] |
| ENSRNOT00000011044 | 621,9875 | -2,61757 | 0,260973 | 1,2E-25 | 1,08E-05 | protein_coding | Wnt5b | Wnt family member 5B [Source:RGD Symbol;Acc:628850] |
| ENSRNOT00000014033 | 3158,465 | -2,61836 | 0,233641 | 4,47E-31 | 1,79E-07 | protein_coding | Tns2 | tensin 2 [Source:RGD Symbol;Acc:1310917] |
| ENSRNOT00000048535 | 127,4828 | -2,61916 | 0,27972 | 6,59E-23 | 4,62E-06 | protein_coding | RT1-CE3 | RT1 class I, locus CE3 [Source:RGD Symbol;Acc:1598231] |
| ENSRNOT00000089378 | 142,7075 | -2,64567 | 0,299715 | 1,24E-20 | 0,000198 | protein_coding | Cfap73 | cilia and flagella associated protein 73 [Source:RGD Symbol;Acc:1561303] |
| ENSRNOT00000027022 | 127,6824 | -2,64583 | 0,280247 | 4,48E-23 | 2,91E-07 | protein_coding | Doc2a | double C2 domain alpha [Source:RGD Symbol;Acc:620518] |
| ENSRNOT00000001051 | 222,9448 | -2,66727 | 0,226173 | 5,7E-34 | 2,8E-12 | protein_coding | Tbc1d32 | TBC1 domain family, member 32 [Source:RGD Symbol;Acc:2323599] |
| ENSRNOT00000071482 | 148,0363 | -2,66824 | 0,306167 | 4,09E-20 | 0,002215 | protein_coding | Tor1aip2 | torsin 1A interacting protein 2 [Source:RGD Symbol;Acc:735059] |
| ENSRNOT00000102788 | 317,3153 | -2,67167 | 0,280074 | 2,07E-23 | 4,06E-07 | protein_coding | Mrps11 | mitochondrial ribosomal protein S11 [Source:RGD Symbol;Acc:1559901] |
| ENSRNOT00000085152 | 335,9752 | -2,67867 | 0,239243 | 6,45E-31 | 3,09E-12 | protein_coding | Kifc2 | kinesin family member C2 [Source:RGD Symbol;Acc:735021] |
| ENSRNOT00000048735 | 687,6715 | -2,67954 | 0,265523 | 5,8E-26 | 6,01E-06 | protein_coding | AABR07006688.1 | |
| ENSRNOT00000023483 | 229,4309 | -2,68312 | 0,237916 | 2,8E-31 | 8,35E-10 | protein_coding | AC103221.1 | |
| ENSRNOT00000048323 | 270,8013 | -2,68618 | 0,232089 | 7,11E-33 | 3,25E-14 | protein_coding | RGD1563667 | similar to TDPOZ3 [Source:RGD Symbol;Acc:1563667] |
| ENSRNOT00000077201 | 162,4533 | -2,69621 | 0,313284 | 9,43E-20 | 0,000372 | protein_coding | Trim15 | tripartite motif containing 15 [Source:RGD Symbol;Acc:6496746] |
| ENSRNOT00000056706 | 3274,521 | -2,69652 | 0,176348 | 1,14E-54 | 5,83E-15 | protein_coding | Eml6 | EMAP like 6 [Source:RGD Symbol;Acc:1561672] |
| ENSRNOT00000076382 | 492,9327 | -2,70088 | 0,282366 | 1,34E-23 | 2,32E-05 | protein_coding | RGD1561796 | RGD1561796 [Source:RGD Symbol;Acc:1561796] |
| ENSRNOT00000081952 | 1532,338 | -2,70587 | 0,244012 | 1,68E-30 | 1,81E-05 | protein_coding | Rab6b | RAB6B, member RAS oncogene family [Source:RGD Symbol;Acc:1309958] |
| ENSRNOT00000010246 | 1792,17 | -2,71814 | 0,255614 | 2,03E-28 | 3,88E-06 | protein_coding | Gcc1 | GRIP and coiled-coil domain containing 1 [Source:RGD Symbol;Acc:2319954] |
| ENSRNOT00000109673 | 471,614 | -2,72547 | 0,297784 | 5,98E-22 | 0,00103 | protein_coding | Arhgef33 | Rho guanine nucleotide exchange factor 33 [Source:RGD Symbol;Acc:1560224] |
| ENSRNOT00000011936 | 112,803 | -2,73177 | 0,324439 | 4,11E-19 | 0,00086 | protein_coding | Fgf22 | fibroblast growth factor 22 [Source:RGD Symbol;Acc:620177] |
| ENSRNOT00000112384 | 132,9892 | -2,73352 | 0,276312 | 4,08E-25 | 9,32E-06 | protein_coding | Ddx42 | DEAD-box helicase 42 [Source:RGD Symbol;Acc:1304909] |
| ENSRNOT00000000107 | 355,1238 | -2,73371 | 0,269002 | 2,29E-26 | 6,78E-06 | protein_coding | Pdzk1 | PDZ domain containing 1 [Source:RGD Symbol;Acc:70924] |
| ENSRNOT00000104580 | 174,802 | -2,73462 | 0,357132 | 2,24E-16 | 0,006128 | protein_coding | Eme1 | essential meiotic structure-specific endonuclease 1 [Source:RGD Symbol;Acc:1309067] |
| ENSRNOT00000003438 | 432,6998 | -2,74709 | 0,198072 | 1,41E-45 | 5,83E-15 | protein_coding | Gpr137b | G protein-coupled receptor 137B [Source:RGD Symbol;Acc:1307342] |
| ENSRNOT00000016479 | 312,2179 | -2,75342 | 0,212671 | 3,22E-40 | 5,83E-15 | protein_coding | Slirp | SRA stem-loop interacting RNA binding protein [Source:RGD Symbol;Acc:1585290] |
| ENSRNOT00000115059 | 112,8847 | -2,75699 | 0,307582 | 3,56E-21 | 0,001962 | protein_coding | Tubgcp4 | tubulin, gamma complex associated protein 4 [Source:RGD Symbol;Acc:1306924] |
| ENSRNOT00000119446 | 527,7231 | -2,75799 | 0,280691 | 8,41E-25 | 5,81E-05 | protein_coding | Ggnbp1 | gametogenetin binding protein 1 [Source:RGD Symbol;Acc:1359729] |
| ENSRNOT00000118739 | 529,5814 | -2,75887 | 0,248534 | 1,45E-30 | 1,4E-06 | protein_coding | Cmtm3 | CKLF-like MARVEL transmembrane domain containing 3 [Source:RGD Symbol;Acc:1308763] |
| ENSRNOT00000028475 | 711,1276 | -2,79121 | 0,256865 | 2,26E-29 | 1,24E-06 | protein_coding | Mrpl49 | mitochondrial ribosomal protein L49 [Source:RGD Symbol;Acc:1309920] |
| ENSRNOT00000007721 | 249,6854 | -2,7928 | 0,267241 | 1,24E-27 | 2,16E-06 | protein_coding | Kif5a | kinesin family member 5A [Source:RGD Symbol;Acc:1303035] |
| ENSRNOT00000026601 | 1251,752 | -2,79411 | 0,192512 | 1,3E-49 | 5,83E-15 | protein_coding | Mk1 | Mk1 protein [Source:RGD Symbol;Acc:621165] |
| ENSRNOT00000110738 | 2668,879 | -2,80096 | 0,302494 | 2,1E-22 | 0,000146 | protein_coding | Gps2 | G protein pathway suppressor 2 [Source:RGD Symbol;Acc:1562746] |
| ENSRNOT00000080446 | 330,7059 | -2,80503 | 0,409722 | 1,15E-13 | 0,008715 | protein_coding | Spata22 | spermatogenesis associated 22 [Source:RGD Symbol;Acc:1565378] |
| ENSRNOT00000009080 | 319,5248 | -2,80908 | 0,317458 | 9,33E-21 | 0,000283 | protein_coding | Baz1a | bromodomain adjacent to zinc finger domain, 1A [Source:RGD Symbol;Acc:1306199] |
| ENSRNOT00000113299 | 248,3022 | -2,81476 | 0,22328 | 2,98E-38 | 8,62E-12 | protein_coding | Tasor | transcription activation suppressor [Source:RGD Symbol;Acc:1595469] |
| ENSRNOT00000116117 | 117,6079 | -2,82702 | 0,300529 | 5,06E-23 | 5,03E-05 | protein_coding | Rmc1 | regulator of MON1-CCZ1 [Source:RGD Symbol;Acc:1311805] |
| ENSRNOT00000113035 | 108,7878 | -2,82926 | 0,333906 | 2,57E-19 | 0,002282 | protein_coding | Gm17949 | adenylate kinase isoenzyme 5 [Source:RGD Symbol;Acc:150345221] |
| ENSRNOT00000044844 | 574,5922 | -2,83308 | 0,230647 | 1,46E-36 | 6,87E-12 | protein_coding | Ldhd | lactate dehydrogenase D [Source:RGD Symbol;Acc:1308107] |
| ENSRNOT00000118879 | 574,1695 | -2,83388 | 0,190367 | 6,03E-52 | 5,83E-15 | protein_coding | Serpinb6a | serpin family B member 6A [Source:RGD Symbol;Acc:735108] |
| ENSRNOT00000051685 | 274,8492 | -2,8358 | 0,28105 | 7,37E-26 | 1,36E-05 | protein_coding | Zfp839 | zinc finger protein 839 [Source:RGD Symbol;Acc:1562557] |
| ENSRNOT00000017000 | 213,1433 | -2,83584 | 0,317851 | 4,43E-21 | 0,007191 | protein_coding | S100a1 | S100 calcium binding protein A1 [Source:RGD Symbol;Acc:3614] |
| ENSRNOT00000085766 | 633,1498 | -2,83799 | 0,275606 | 7,23E-27 | 3,11E-05 | protein_coding | Ogg1 | 8-oxoguanine DNA glycosylase [Source:RGD Symbol;Acc:621168] |
| ENSRNOT00000095552 | 548,0859 | -2,84317 | 0,321759 | 1,13E-20 | 3,04E-05 | protein_coding | B3gnt4 | UDP-GlcNAc:betaGal beta-1,3-N-acetylglucosaminyltransferase 4 [Source:RGD Symbol;Acc:1309612] |
| ENSRNOT00000004896 | 554,276 | -2,8463 | 0,297747 | 1,62E-23 | 0,000457 | protein_coding | Ppox | protoporphyrinogen oxidase [Source:RGD Symbol;Acc:1310543] |
| ENSRNOT00000115646 | 323,5532 | -2,84661 | 0,237601 | 4,87E-35 | 6,9E-09 | protein_coding | Hic1 | HIC ZBTB transcriptional repressor 1 [Source:RGD Symbol;Acc:1307527] |
| ENSRNOT00000105687 | 596,3884 | -2,84959 | 0,239528 | 1,51E-34 | 7,8E-08 | protein_coding | Sh3gl1 | SH3 domain containing GRB2 like 1, endophilin A2 [Source:RGD Symbol;Acc:708456] |
| ENSRNOT00000068124 | 346,0156 | -2,85739 | 0,307418 | 1,48E-22 | 6,11E-06 | protein_coding | Kbtbd4 | kelch repeat and BTB domain containing 4 [Source:RGD Symbol;Acc:1310234] |
| ENSRNOT00000001536 | 462,1465 | -2,85968 | 0,281914 | 2,76E-26 | 3,2E-06 | protein_coding | Srsf9 | serine and arginine rich splicing factor 9 [Source:RGD Symbol;Acc:1309495] |
| ENSRNOT00000113703 | 254,4038 | -2,87819 | 0,243704 | 4,87E-34 | 1,08E-12 | protein_coding | Klhdc2 | kelch domain containing 2 [Source:RGD Symbol;Acc:1306504] |
| ENSRNOT00000076833 | 903,8218 | -2,88217 | 0,263135 | 7,36E-30 | 1,39E-07 | protein_coding | Znfx1 | zinc finger, NFX1-type containing 1 [Source:RGD Symbol;Acc:1306344] |
| ENSRNOT00000099043 | 268,2568 | -2,88375 | 0,264838 | 1,52E-29 | 0,000148 | protein_coding | Lrrc75a | leucine rich repeat containing 75A [Source:RGD Symbol;Acc:1595947] |
| ENSRNOT00000015878 | 10498,7 | -2,88861 | 0,26828 | 7,24E-29 | 7,52E-07 | protein_coding | Tifab | TIFA inhibitor [Source:RGD Symbol;Acc:1311681] |
| ENSRNOT00000101849 | 379,3836 | -2,91401 | 0,228682 | 5,31E-39 | 2,18E-13 | protein_coding | Syngr3 | synaptogyrin 3 [Source:RGD Symbol;Acc:1311801] |
| ENSRNOT00000094347 | 1292,432 | -2,92326 | 0,234768 | 2,01E-37 | 6,34E-10 | protein_coding | Plppr3 | phospholipid phosphatase related 3 [Source:RGD Symbol;Acc:727823] |
| ENSRNOT00000005370 | 7983,059 | -2,92729 | 0,23691 | 6,94E-37 | 1,11E-08 | protein_coding | Pfn1 | profilin 1 [Source:RGD Symbol;Acc:621825] |
| ENSRNOT00000020147 | 288,7495 | -2,92776 | 0,275436 | 2,64E-28 | 4,75E-07 | protein_coding | Lrrc71 | leucine rich repeat containing 71 [Source:RGD Symbol;Acc:1309453] |
| ENSRNOT00000029359 | 181,3976 | -2,93222 | 0,318952 | 3,74E-22 | 0,000179 | protein_coding | Tvp23a | trans-golgi network vesicle protein 23 homolog A [Source:RGD Symbol;Acc:1566405] |
| ENSRNOT00000065115 | 595,5677 | -2,93353 | 0,23313 | 4,74E-38 | 5,83E-15 | protein_coding | LOC499643 | similar to hypothetical protein FLJ25371 [Source:RGD Symbol;Acc:1566255] |
| ENSRNOT00000095720 | 939,1664 | -2,93891 | 0,272533 | 4,42E-29 | 3,1E-05 | protein_coding | Ints9 | integrator complex subunit 9 [Source:RGD Symbol;Acc:1311539] |
| ENSRNOT00000047286 | 4872,301 | -2,93994 | 0,302848 | 2,53E-24 | 0,000244 | protein_coding | Zfp341 | zinc finger protein 341 [Source:RGD Symbol;Acc:1564726] |
| ENSRNOT00000042117 | 180,7601 | -2,94106 | 0,300132 | 1,24E-24 | 0,000318 | protein_coding | Pdgfa | platelet derived growth factor subunit A [Source:RGD Symbol;Acc:3282] |
| ENSRNOT00000093879 | 218,9318 | -2,94832 | 0,304309 | 4,06E-24 | 0,000229 | protein_coding | Fastk | Fas-activated serine/threonine kinase [Source:RGD Symbol;Acc:1311601] |
| ENSRNOT00000016177 | 697,9902 | -2,96814 | 0,262242 | 1,87E-31 | 2,09E-11 | protein_coding | Sox15 | SRY-box transcription factor 15 [Source:RGD Symbol;Acc:1312022] |
| ENSRNOT00000108247 | 350,5983 | -2,9701 | 0,220651 | 1,56E-43 | 5,83E-15 | protein_coding | Fbxl13 | F-box and leucine-rich repeat protein 13 [Source:RGD Symbol;Acc:1642425] |
| ENSRNOT00000097960 | 1531,834 | -2,97102 | 0,312664 | 3,1E-23 | 0,000152 | protein_coding | Pip4k2b | phosphatidylinositol-5-phosphate 4-kinase type 2 beta [Source:RGD Symbol;Acc:621710] |
| ENSRNOT00000034670 | 125,1274 | -2,97274 | 0,328754 | 1,5E-21 | 0,000115 | protein_coding | Tpte2 | transmembrane phosphoinositide 3-phosphatase and tensin homolog 2 [Source:RGD Symbol;Acc:1305825] |
| ENSRNOT00000028097 | 344,2695 | -2,97726 | 0,306203 | 1,88E-24 | 0,000134 | protein_coding | Rnaseh2c | ribonuclease H2, subunit C [Source:RGD Symbol;Acc:2319141] |
| ENSRNOT00000098589 | 458,9611 | -2,9774 | 0,270089 | 3,56E-30 | 1,2E-11 | protein_coding | Bbof1 | basal body orientation factor 1 [Source:RGD Symbol;Acc:1560978] |
| ENSRNOT00000068105 | 225,7576 | -2,97807 | 0,267668 | 1,07E-30 | 2,47E-08 | protein_coding | Msmp | microseminoprotein, prostate associated [Source:RGD Symbol;Acc:2323536] |
| ENSRNOT00000078980 | 147,1268 | -2,97827 | 0,24745 | 2,15E-35 | 3,65E-12 | protein_coding | Morc2 | MORC family CW-type zinc finger 2 [Source:RGD Symbol;Acc:1310842] |
| ENSRNOT00000075273 | 1284,798 | -2,97945 | 0,247139 | 1,66E-35 | 1,33E-09 | protein_coding | Smim10l1 | small integral membrane protein 10 like 1 [Source:RGD Symbol;Acc:2322729] |
| ENSRNOT00000097234 | 256,7135 | -2,99538 | 0,255808 | 1,45E-33 | 6,69E-09 | protein_coding | Tmem208 | transmembrane protein 208 [Source:RGD Symbol;Acc:1560953] |
| ENSRNOT00000039306 | 355,0926 | -2,99861 | 0,271358 | 2,3E-30 | 2,6E-08 | protein_coding | Nemp1 | nuclear envelope integral membrane protein 1 [Source:RGD Symbol;Acc:1587179] |
| ENSRNOT00000039216 | 185,2333 | -2,99989 | 0,279407 | 1,02E-28 | 5,53E-07 | protein_coding | Zscan30 | zinc finger and SCAN domain containing 30 [Source:RGD Symbol;Acc:1306095] |
| ENSRNOT00000012794 | 206,8644 | -3,00323 | 0,252504 | 1,77E-34 | 2,61E-11 | protein_coding | Bricd5 | BRICHOS domain containing 5 [Source:RGD Symbol;Acc:1596720] |
| ENSRNOT00000119157 | 130,5017 | -3,00491 | 0,278307 | 3,8E-29 | 8,59E-07 | protein_coding | Dimt1 | DIM1 rRNA methyltransferase and ribosome maturation factor [Source:RGD Symbol;Acc:1311752] |
| ENSRNOT00000106970 | 530,0774 | -3,006 | 0,242104 | 4,35E-37 | 5,2E-13 | protein_coding | Csf2ra | colony stimulating factor 2 receptor subunit alpha [Source:RGD Symbol;Acc:1594330] |
| **ENSRNOT00000051371** | **190,5887** | **-3,01077** | **0,23697** | **1,03E-38** | **4,21E-13** | **protein_coding** | **Cacna1d** | **calcium voltage-gated channel subunit alpha1 D [Source:RGD Symbol;Acc:70973]** |
| **ENSRNOT00000089074** | **578,1755** | **-3,01407** | **0,277758** | **2,56E-29** | **0,000566** | **protein_coding** | **Myl6** | **myosin light chain 6 [Source:RGD Symbol;Acc:1589019]** |
| ENSRNOT00000005352 | 107,7746 | -3,01596 | 0,349648 | 8,7E-20 | 0,000738 | protein_coding | Klhdc9 | kelch domain containing 9 [Source:RGD Symbol;Acc:1561025] |
| ENSRNOT00000060311 | 337,1669 | -3,01613 | 0,224862 | 3,2E-43 | 1,01E-10 | protein_coding | Tcp11l1 | t-complex 11 like 1 [Source:RGD Symbol;Acc:1562313] |
| ENSRNOT00000071548 | 914,9656 | -3,01969 | 0,19302 | 6,14E-57 | 5,83E-15 | protein_coding | Zfp397 | zinc finger protein 397 [Source:RGD Symbol;Acc:6504280] |
| ENSRNOT00000090916 | 170,9577 | -3,02494 | 0,341022 | 6,77E-21 | 5,03E-05 | protein_coding | Cirop | ciliated left-right organizer metallopeptidase [Source:RGD Symbol;Acc:1560492] |
| ENSRNOT00000106983 | 177,4012 | -3,02593 | 0,268093 | 2,52E-31 | 1,37E-10 | protein_coding | Rpl31 | ribosomal protein L31 [Source:RGD Symbol;Acc:621202] |
| ENSRNOT00000106741 | 666,7291 | -3,02983 | 0,237844 | 6,26E-39 | 2,72E-14 | protein_coding | Macrod1 | mono-ADP ribosylhydrolase 1 [Source:RGD Symbol;Acc:628701] |
| ENSRNOT00000061904 | 133,0616 | -3,03123 | 0,282777 | 1,36E-28 | 1,73E-09 | protein_coding | C8g | complement C8 gamma chain [Source:RGD Symbol;Acc:1309074] |
| ENSRNOT00000095390 | 306,924 | -3,03429 | 0,332423 | 8,68E-22 | 0,000457 | protein_coding | Cnbd2 | cyclic nucleotide binding domain containing 2 [Source:RGD Symbol;Acc:1311678] |
| ENSRNOT00000091554 | 507,2075 | -3,03759 | 0,267853 | 1,38E-31 | 0,000318 | protein_coding | Prpf18 | pre-mRNA processing factor 18 [Source:RGD Symbol;Acc:708550] |
| ENSRNOT00000105902 | 332,9876 | -3,03985 | 0,400443 | 4,25E-16 | 4,75E-05 | protein_coding | Slc16a13 | solute carrier family 16, member 13 [Source:RGD Symbol;Acc:1359138] |
| ENSRNOT00000111911 | 160,0324 | -3,04885 | 0,301383 | 4,36E-26 | 0,000103 | protein_coding | Cxcr6 | C-X-C motif chemokine receptor 6 [Source:RGD Symbol;Acc:1642118] |
| ENSRNOT00000036586 | 957,3109 | -3,05531 | 0,215457 | 1,96E-47 | 5,83E-15 | protein_coding | Serhl2 | serine hydrolase-like 2 [Source:RGD Symbol;Acc:1563386] |
| ENSRNOT00000114142 | 326,243 | -3,05629 | 0,25028 | 4,06E-36 | 7,19E-11 | protein_coding | Prrx2 | paired related homeobox 2 [Source:RGD Symbol;Acc:1311471] |
| ENSRNOT00000027086 | 4330,35 | -3,0602 | 0,192437 | 8,7E-59 | 5,83E-15 | protein_coding | Cnbd2 | cyclic nucleotide binding domain containing 2 [Source:RGD Symbol;Acc:1311678] |
| ENSRNOT00000043724 | 272,5168 | -3,06583 | 0,281343 | 1,13E-29 | 1,12E-07 | protein_coding | Garin5a | golgi associated RAB2 interactor 5A [Source:RGD Symbol;Acc:1311142] |
| ENSRNOT00000085311 | 262,9755 | -3,08507 | 0,302136 | 1,33E-26 | 3,81E-06 | protein_coding | Creb3 | cAMP responsive element binding protein 3 [Source:RGD Symbol;Acc:1308831] |
| ENSRNOT00000026854 | 867,8325 | -3,09148 | 0,192095 | 9,09E-60 | 5,83E-15 | protein_coding | Gmppb | GDP-mannose pyrophosphorylase B [Source:RGD Symbol;Acc:1560458] |
| ENSRNOT00000114762 | 128,348 | -3,09442 | 0,329431 | 5,72E-23 | 8,82E-06 | protein_coding | Recql | RecQ like helicase [Source:RGD Symbol;Acc:1311071] |
| ENSRNOT00000117076 | 99,24406 | -3,09993 | 0,353751 | 2,13E-20 | 0,001962 | protein_coding | Kars1 | lysyl-tRNA synthetase 1 [Source:RGD Symbol;Acc:1359653] |
| ENSRNOT00000011688 | 414,3143 | -3,12102 | 0,203861 | 6,27E-55 | 5,83E-15 | protein_coding | Rars2 | arginyl-tRNA synthetase 2, mitochondrial [Source:RGD Symbol;Acc:1305419] |
| ENSRNOT00000110822 | 320,3813 | -3,12694 | 0,255706 | 3,46E-36 | 2,84E-11 | protein_coding | Tinf2 | TERF1 interacting nuclear factor 2 [Source:RGD Symbol;Acc:1359192] |
| ENSRNOT00000097479 | 1070,695 | -3,12717 | 0,20913 | 1,9E-52 | 5,83E-15 | protein_coding | Ldha | lactate dehydrogenase A [Source:RGD Symbol;Acc:2996] |
| ENSRNOT00000012519 | 470,5941 | -3,12827 | 0,24005 | 9,97E-41 | 2,78E-12 | protein_coding | Mob3b | MOB kinase activator 3B [Source:RGD Symbol;Acc:1560696] |
| ENSRNOT00000079667 | 119,5279 | -3,13274 | 0,319486 | 1,1E-24 | 4,31E-06 | protein_coding | Mcc | MCC regulator of WNT signaling pathway [Source:RGD Symbol;Acc:1561988] |
| ENSRNOT00000000022 | 230,679 | -3,13534 | 0,248111 | 2,09E-38 | 3,43E-11 | protein_coding | Fbxl14-ps1 | F-box and leucine-rich repeat protein 14, pseudogene 1 [Source:RGD Symbol;Acc:11478102] |
| ENSRNOT00000059524 | 1550,96 | -3,15139 | 0,155778 | 2,67E-92 | 5,83E-15 | protein_coding | Abl2 | ABL proto-oncogene 2, non-receptor tyrosine kinase [Source:RGD Symbol;Acc:1590898] |
| ENSRNOT00000078646 | 155,3477 | -3,15585 | 0,330115 | 1,48E-23 | 0,000269 | protein_coding | Ac1576 | uncharacterized LOC102552783 [Source:RGD Symbol;Acc:7502825] |
| ENSRNOT00000094708 | 585,3582 | -3,15657 | 0,207394 | 5,26E-54 | 5,83E-15 | protein_coding | Dgat1 | diacylglycerol O-acyltransferase 1 [Source:RGD Symbol;Acc:628673] |
| ENSRNOT00000112332 | 720,9801 | -3,16105 | 0,265872 | 2,31E-34 | 2,86E-12 | protein_coding | LOC500475 | similar to hypothetical protein 4933430I17 [Source:RGD Symbol;Acc:1563460] |
| ENSRNOT00000017476 | 105,4755 | -3,16196 | 0,291821 | 2,91E-29 | 5,41E-09 | protein_coding | Tox4 | TOX high mobility group box family member 4 [Source:RGD Symbol;Acc:708449] |
| ENSRNOT00000029237 | 1494,876 | -3,16493 | 0,272941 | 6,07E-33 | 7,24E-09 | protein_coding | Tbc1d10c | TBC1 domain family, member 10C [Source:RGD Symbol;Acc:1311490] |
| ENSRNOT00000099813 | 2981,444 | -3,16731 | 0,204095 | 3,72E-56 | 5,83E-15 | protein_coding | Ap1s1 | adaptor related protein complex 1 subunit sigma 1 [Source:RGD Symbol;Acc:1305911] |
| ENSRNOT00000037918 | 19264,83 | -3,17321 | 0,268282 | 4,19E-34 | 6,06E-11 | protein_coding | Syn3 | synapsin III [Source:RGD Symbol;Acc:3799] |
| ENSRNOT00000076880 | 200,6197 | -3,18092 | 0,259834 | 2,98E-36 | 1,79E-11 | protein_coding | Gmps | guanine monophosphate synthase [Source:RGD Symbol;Acc:1310063] |
| ENSRNOT00000100407 | 253,1174 | -3,18468 | 0,300235 | 3,03E-28 | 6,76E-07 | protein_coding | Ccdc167 | coiled-coil domain containing 167 [Source:RGD Symbol;Acc:1588733] |
| ENSRNOT00000064685 | 707,4195 | -3,18536 | 0,215111 | 1,68E-51 | 5,83E-15 | protein_coding | Xpa | XPA, DNA damage recognition and repair factor [Source:RGD Symbol;Acc:1308769] |
| ENSRNOT00000108801 | 370,3602 | -3,19002 | 0,199552 | 4,33E-59 | 5,83E-15 | protein_coding | Gcnt7 | glucosaminyl (N-acetyl) transferase family member 7 [Source:RGD Symbol;Acc:7496421] |
| ENSRNOT00000075641 | 162,8217 | -3,20198 | 0,334431 | 9,6E-24 | 0,000288 | protein_coding | LOC102554231 | paired immunoglobulin-like type 2 receptor alpha-like [Source:RGD Symbol;Acc:7553419] |
| ENSRNOT00000092186 | 778,9059 | -3,20232 | 0,23418 | 1,98E-44 | 2,22E-14 | protein_coding | Adap1 | ArfGAP with dual PH domains 1 [Source:RGD Symbol;Acc:621418] |
| ENSRNOT00000015641 | 129,9732 | -3,20286 | 0,347432 | 2,97E-22 | 0,000149 | protein_coding | Foxp3 | forkhead box P3 [Source:RGD Symbol;Acc:1562112] |
| ENSRNOT00000106750 | 530,2772 | -3,20711 | 0,211617 | 9,14E-54 | 5,83E-15 | protein_coding | B3galt9 | beta-1,3-galactosyltransferase 9 [Source:RGD Symbol;Acc:9424697] |
| ENSRNOT00000034902 | 1094,495 | -3,21058 | 0,19733 | 4,42E-61 | 5,83E-15 | protein_coding | Yars2 | tyrosyl-tRNA synthetase 2 [Source:RGD Symbol;Acc:1311696] |
| ENSRNOT00000033533 | 131,9089 | -3,21956 | 0,338466 | 2,87E-23 | 8,91E-06 | protein_coding | Ppp2r3c | protein phosphatase 2, regulatory subunit B'', gamma [Source:RGD Symbol;Acc:1309207] |
| ENSRNOT00000086305 | 2075,869 | -3,22033 | 0,355856 | 1,34E-21 | 0,005483 | protein_coding | Coro7 | coronin 7 [Source:RGD Symbol;Acc:621591] |
| ENSRNOT00000084410 | 210,2004 | -3,22194 | 0,287806 | 7,15E-31 | 5,17E-09 | protein_coding | Rbm4b | RNA binding motif protein 4B [Source:RGD Symbol;Acc:1359343] |
| ENSRNOT00000064484 | 234,7551 | -3,22248 | 0,254227 | 1,23E-38 | 3,55E-13 | protein_coding | Trmt13 | tRNA methyltransferase 13 homolog [Source:RGD Symbol;Acc:1565211] |
| ENSRNOT00000016387 | 101,2579 | -3,22679 | 0,386827 | 7,56E-19 | 0,006681 | protein_coding | Ap3m1 | adaptor related protein complex 3 subunit mu 1 [Source:RGD Symbol;Acc:620417] |
| ENSRNOT00000113107 | 338,082 | -3,23042 | 0,282901 | 4,52E-32 | 7,08E-11 | protein_coding | Larp1 | La ribonucleoprotein 1, translational regulator [Source:RGD Symbol;Acc:1306683] |
| ENSRNOT00000015975 | 407,4477 | -3,23941 | 0,264201 | 1,79E-36 | 7,86E-14 | protein_coding | AABR07008940.1 | |
| ENSRNOT00000098533 | 453,854 | -3,24055 | 0,284207 | 5,95E-32 | 4,59E-07 | protein_coding | Immp2l | inner mitochondrial membrane peptidase subunit 2 [Source:RGD Symbol;Acc:2323665] |
| ENSRNOT00000111882 | 1155,883 | -3,24683 | 0,201351 | 5,66E-60 | 5,83E-15 | protein_coding | Cbr2 | carbonyl reductase 2 [Source:RGD Symbol;Acc:1590051] |
| ENSRNOT00000092054 | 660,5417 | -3,24811 | 0,327971 | 3E-25 | 0,000417 | protein_coding | Rmdn1 | regulator of microtubule dynamics 1 [Source:RGD Symbol;Acc:1563085] |
| ENSRNOT00000023914 | 1283,924 | -3,25205 | 0,233576 | 1,07E-45 | 1,11E-11 | protein_coding | Bphl | biphenyl hydrolase like [Source:RGD Symbol;Acc:1307572] |
| ENSRNOT00000096738 | 2743,115 | -3,25308 | 0,249595 | 7,58E-41 | 5,99E-09 | protein_coding | Eno3 | enolase 3 [Source:RGD Symbol;Acc:2555] |
| ENSRNOT00000027645 | 456,0007 | -3,25498 | 0,211063 | 1,56E-55 | 5,83E-15 | protein_coding | Capn12 | calpain 12 [Source:RGD Symbol;Acc:1307341] |
| ENSRNOT00000109940 | 206,4202 | -3,25707 | 0,271182 | 3,93E-35 | 1,29E-10 | protein_coding | Tlk1 | tousled-like kinase 1 [Source:RGD Symbol;Acc:1310370] |
| ENSRNOT00000105139 | 2702,271 | -3,25832 | 0,261707 | 2,35E-37 | 2,41E-09 | protein_coding | Surf2 | surfeit 2 [Source:RGD Symbol;Acc:1565817] |
| ENSRNOT00000026067 | 236,989 | -3,26275 | 0,228034 | 3,69E-48 | 5,83E-15 | protein_coding | Dkk4 | dickkopf WNT signaling pathway inhibitor 4 [Source:RGD Symbol;Acc:1563172] |
| ENSRNOT00000108772 | 629,7771 | -3,27203 | 0,237588 | 5,7E-45 | 5,83E-15 | protein_coding | LOC100125364 | RIKEN cDNA 1600012H06 gene [Source:MGI Symbol;Acc:MGI:1915162] |
| ENSRNOT00000111317 | 382,9448 | -3,27652 | 0,252929 | 2,51E-40 | 7,2E-12 | protein_coding | Nit1 | nitrilase 1 [Source:RGD Symbol;Acc:727821] |
| ENSRNOT00000004499 | 606,265 | -3,28275 | 0,213008 | 2,37E-55 | 5,83E-15 | protein_coding | Mcm9 | minichromosome maintenance 9 homologous recombination repair factor [Source:RGD Symbol;Acc:1560557] |
| ENSRNOT00000098915 | 316,4144 | -3,28436 | 0,278782 | 6,66E-34 | 6,66E-08 | protein_coding | Cops6 | COP9 signalosome subunit 6 [Source:RGD Symbol;Acc:1309919] |
| ENSRNOT00000070979 | 576,2871 | -3,28724 | 0,285748 | 1,45E-32 | 6,69E-09 | protein_coding | Cenpo | centromere protein O [Source:RGD Symbol;Acc:1590328] |
| ENSRNOT00000104746 | 196,6031 | -3,3088 | 0,266041 | 3,44E-37 | 4,81E-14 | protein_coding | LOC103692302 | uncharacterized LOC103692302 [Source:RGD Symbol;Acc:9116417] |
| ENSRNOT00000035057 | 233,9596 | -3,30913 | 0,294246 | 3,85E-31 | 4,12E-10 | protein_coding | RGD1559622 | similar to hypothetical protein C130079G13 [Source:RGD Symbol;Acc:1559622] |
| ENSRNOT00000111632 | 5594,439 | -3,31267 | 0,186776 | 5,21E-72 | 5,83E-15 | protein_coding | Septin11 | septin 11 [Source:RGD Symbol;Acc:1307405] |
| ENSRNOT00000061194 | 391,5348 | -3,31481 | 0,273803 | 1,02E-35 | 4,53E-09 | protein_coding | Tektip1 | tektin bundle interacting protein 1 [Source:RGD Symbol;Acc:1593449] |
| ENSRNOT00000102516 | 1148,612 | -3,31699 | 0,238164 | 7,51E-46 | 5,83E-15 | protein_coding | Tmem179b | transmembrane protein 179B [Source:RGD Symbol;Acc:1582850] |
| ENSRNOT00000088716 | 102,4287 | -3,32103 | 0,311654 | 1,73E-28 | 6,18E-07 | protein_coding | Plppr1 | phospholipid phosphatase related 1 [Source:RGD Symbol;Acc:1303116] |
| ENSRNOT00000107762 | 273,8827 | -3,32564 | 0,342697 | 2,88E-24 | 1,79E-07 | protein_coding | Ikbkg | inhibitor of nuclear factor kappa B kinase regulatory subunit gamma [Source:RGD Symbol;Acc:735223] |
| ENSRNOT00000112591 | 799,6156 | -3,32627 | 0,213055 | 1,23E-56 | 5,83E-15 | protein_coding | Gipc1 | GIPC PDZ domain containing family, member 1 [Source:RGD Symbol;Acc:68338] |
| ENSRNOT00000068003 | 826,9328 | -3,33643 | 0,341547 | 1,59E-24 | 0,001486 | protein_coding | Mfsd4a | major facilitator superfamily domain containing 4A [Source:RGD Symbol;Acc:1566090] |
| ENSRNOT00000005685 | 388,2835 | -3,34879 | 0,273434 | 2,57E-36 | 4,22E-09 | protein_coding | Gpr182 | G protein-coupled receptor 182 [Source:RGD Symbol;Acc:61903] |
| ENSRNOT00000110610 | 214,3671 | -3,34902 | 0,365693 | 5,38E-22 | 0,001738 | protein_coding | Hpf1 | histone PARylation factor 1 [Source:RGD Symbol;Acc:1311747] |
| ENSRNOT00000097525 | 588,4257 | -3,35758 | 0,230834 | 8,44E-50 | 5,83E-15 | protein_coding | Zfp668 | zinc finger protein 668 [Source:RGD Symbol;Acc:1308623] |
| ENSRNOT00000094623 | 268,6655 | -3,36165 | 0,265397 | 1,47E-38 | 8,84E-12 | protein_coding | Fubp1 | far upstream element binding protein 1 [Source:RGD Symbol;Acc:1591892] |
| ENSRNOT00000103816 | 480,9977 | -3,36202 | 0,289125 | 3,92E-33 | 6,54E-06 | protein_coding | Ccr9 | C-C motif chemokine receptor 9 [Source:RGD Symbol;Acc:628750] |
| ENSRNOT00000112238 | 462,4211 | -3,36487 | 0,252091 | 1,31E-42 | 6,74E-11 | protein_coding | Nab2 | Ngfi-A binding protein 2 [Source:RGD Symbol;Acc:1311712] |
| ENSRNOT00000108514 | 132,7437 | -3,36643 | 0,372819 | 1,68E-21 | 0,000294 | protein_coding | Zfp689 | zinc finger protein 689 [Source:RGD Symbol;Acc:628696] |
| ENSRNOT00000045763 | 436,944 | -3,37006 | 0,237727 | 2,47E-47 | 5,83E-15 | protein_coding | Spats1 | spermatogenesis associated, serine-rich 1 [Source:RGD Symbol;Acc:631367] |
| ENSRNOT00000095761 | 635,5784 | -3,37027 | 0,227124 | 1,02E-51 | 5,83E-15 | protein_coding | Tomm22 | translocase of outer mitochondrial membrane 22 [Source:RGD Symbol;Acc:1303260] |
| ENSRNOT00000094381 | 1221,501 | -3,3841 | 0,206635 | 5,82E-62 | 5,83E-15 | protein_coding | Pea15 | proliferation and apoptosis adaptor protein 15 [Source:RGD Symbol;Acc:1306055] |
| ENSRNOT00000085865 | 1657,365 | -3,38981 | 0,370611 | 6,61E-22 | 0,003399 | protein_coding | AC130391.3 | |
| ENSRNOT00000076528 | 156,1758 | -3,39242 | 0,298567 | 9,77E-32 | 1,21E-08 | protein_coding | Csnk2b | casein kinase 2 beta [Source:RGD Symbol;Acc:619978] |
| ENSRNOT00000093960 | 1556,56 | -3,39912 | 0,211415 | 1,18E-59 | 5,83E-15 | protein_coding | Smyd1 | SET and MYND domain containing 1 [Source:RGD Symbol;Acc:1305105] |
| ENSRNOT00000100088 | 139,1842 | -3,39944 | 0,296585 | 2,49E-32 | 4,48E-13 | protein_coding | Aak1 | AP2 associated kinase 1 [Source:RGD Symbol;Acc:1305520] |
| ENSRNOT00000015864 | 237,5104 | -3,39982 | 0,244142 | 9,07E-46 | 5,83E-15 | protein_coding | Mrpl47 | mitochondrial ribosomal protein L47 [Source:RGD Symbol;Acc:1307531] |
| ENSRNOT00000060492 | 2964,486 | -3,40322 | 0,384345 | 8,42E-21 | 0,002482 | protein_coding | Ccdc152 | coiled-coil domain containing 152 [Source:RGD Symbol;Acc:1561503] |
| ENSRNOT00000103724 | 915,706 | -3,40507 | 0,22355 | 2,39E-54 | 5,83E-15 | protein_coding | Psmc3 | proteasome 26S subunit, ATPase 3 [Source:RGD Symbol;Acc:61905] |
| ENSRNOT00000109943 | 641,5602 | -3,40589 | 0,263889 | 7,04E-40 | 1,21E-10 | protein_coding | Aktip | AKT interacting protein [Source:RGD Symbol;Acc:1311170] |
| ENSRNOT00000067141 | 299,2196 | -3,4069 | 0,326602 | 1,95E-27 | 7,4E-05 | protein_coding | Map1lc3b | microtubule-associated protein 1 light chain 3 beta [Source:RGD Symbol;Acc:621315] |
| ENSRNOT00000092487 | 240,8448 | -3,40857 | 0,322844 | 5,27E-28 | 2,62E-06 | protein_coding | Bri3 | brain protein I3 [Source:RGD Symbol;Acc:1309187] |
| ENSRNOT00000015033 | 1744,504 | -3,41761 | 0,1551 | 2,2E-108 | 5,83E-15 | protein_coding | Rims3 | regulating synaptic membrane exocytosis 3 [Source:RGD Symbol;Acc:628762] |
| ENSRNOT00000019571 | 4025,187 | -3,43159 | 0,225449 | 3,83E-54 | 5,83E-15 | protein_coding | Retsat | retinol saturase [Source:RGD Symbol;Acc:628802] |
| ENSRNOT00000114668 | 592,5775 | -3,43733 | 0,306849 | 5,76E-31 | 4,22E-06 | protein_coding | Tmc6 | transmembrane channel like 6 [Source:RGD Symbol;Acc:1591146] |
| ENSRNOT00000119507 | 193,4698 | -3,43911 | 0,302054 | 6,81E-32 | 1,25E-09 | protein_coding | Sumo2 | small ubiquitin-like modifier 2 [Source:RGD Symbol;Acc:621761] |
| ENSRNOT00000007795 | 1400,434 | -3,44041 | 0,235367 | 3,19E-50 | 5,83E-15 | protein_coding | Nek10 | NIMA-related kinase 10 [Source:RGD Symbol;Acc:1564342] |
| ENSRNOT00000055219 | 129,591 | -3,45392 | 0,294208 | 1,04E-33 | 2,02E-12 | protein_coding | Mroh8 | maestro heat-like repeat family member 8 [Source:RGD Symbol;Acc:2321481] |
| ENSRNOT00000097213 | 981,2588 | -3,45507 | 0,247844 | 4,63E-46 | 5,83E-15 | protein_coding | Uba52 | ubiquitin A-52 residue ribosomal protein fusion product 1 [Source:RGD Symbol;Acc:68344] |
| ENSRNOT00000079015 | 219,9638 | -3,45971 | 0,293939 | 7,75E-34 | 2,05E-08 | protein_coding | Ppia | peptidylprolyl isomerase A [Source:RGD Symbol;Acc:3372] |
| ENSRNOT00000095430 | 290,2725 | -3,46415 | 0,268668 | 8,4E-40 | 1,16E-14 | protein_coding | Vopp1 | VOPP1 WW domain binding protein [Source:RGD Symbol;Acc:1306494] |
| ENSRNOT00000095345 | 161,4486 | -3,46893 | 0,277634 | 8,46E-38 | 1,06E-12 | protein_coding | Gemin4 | gem (nuclear organelle) associated protein 4 [Source:RGD Symbol;Acc:1563715] |
| ENSRNOT00000085562 | 269,3504 | -3,47249 | 0,272067 | 2,77E-39 | 1,42E-10 | protein_coding | Ciao1 | cytosolic iron-sulfur assembly component 1 [Source:RGD Symbol;Acc:1307285] |
| ENSRNOT00000104672 | 906,3821 | -3,47342 | 0,252889 | 9,54E-45 | 8,33E-14 | protein_coding | Tnfaip8l1 | TNF alpha induced protein 8 like 1 [Source:RGD Symbol;Acc:1588574] |
| ENSRNOT00000064245 | 231,4364 | -3,47711 | 0,345969 | 9,41E-26 | 3,07E-05 | protein_coding | Tulp2 | TUB like protein 2 [Source:RGD Symbol;Acc:1310551] |
| ENSRNOT00000029546 | 105,5812 | -3,48568 | 0,368241 | 4,06E-23 | 7,53E-08 | protein_coding | Rps25 | ribosomal protein s25 [Source:RGD Symbol;Acc:621043] |
| ENSRNOT00000024222 | 143,1843 | -3,489 | 0,391372 | 4,94E-21 | 0,000249 | protein_coding | Cfap100 | cilia and flagella associated protein 100 [Source:RGD Symbol;Acc:1305768] |
| ENSRNOT00000066742 | 154,5696 | -3,49566 | 0,342087 | 1,09E-26 | 1,98E-07 | protein_coding | AABR07073146.1 | |
| ENSRNOT00000116214 | 1253,484 | -3,49793 | 0,286643 | 4,71E-36 | 7,28E-11 | protein_coding | U2af1l4 | U2 small nuclear RNA auxiliary factor 1-like 4 [Source:RGD Symbol;Acc:1305600] |
| ENSRNOT00000011303 | 163,7496 | -3,50255 | 0,339523 | 5,31E-27 | 1,11E-06 | protein_coding | Mettl5 | methyltransferase 5, N6-adenosine [Source:RGD Symbol;Acc:1566062] |
| ENSRNOT00000120021 | 517,5675 | -3,50289 | 0,207706 | 3,09E-65 | 5,83E-15 | protein_coding | Commd1 | copper metabolism domain containing 1 [Source:RGD Symbol;Acc:1311771] |
| ENSRNOT00000001153 | 124,2138 | -3,50303 | 0,383212 | 7,93E-22 | 0,00693 | protein_coding | Vwa7 | von Willebrand factor A domain containing 7 [Source:RGD Symbol;Acc:1303137] |
| ENSRNOT00000073239 | 2192,052 | -3,50654 | 0,309479 | 1,69E-31 | 3,39E-07 | protein_coding | Nlrc5 | NLR family, CARD domain containing 5 [Source:RGD Symbol;Acc:1309166] |
| ENSRNOT00000007932 | 789,274 | -3,50926 | 0,247305 | 1,48E-47 | 1,69E-14 | protein_coding | Irak4 | interleukin-1 receptor-associated kinase 4 [Source:RGD Symbol;Acc:1305303] |
| ENSRNOT00000110050 | 246,2188 | -3,51209 | 0,24683 | 1,05E-47 | 5,83E-15 | protein_coding | Chrnb1 | cholinergic receptor nicotinic beta 1 subunit [Source:RGD Symbol;Acc:2349] |
| ENSRNOT00000104796 | 791,4904 | -3,52 | 0,22368 | 1,7E-57 | 5,83E-15 | protein_coding | Btd | biotinidase [Source:RGD Symbol;Acc:1305316] |
| ENSRNOT00000110420 | 640,6584 | -3,5207 | 0,310662 | 1,54E-31 | 5,93E-09 | protein_coding | Adat1 | adenosine deaminase, tRNA-specific 1 [Source:RGD Symbol;Acc:1591020] |
| ENSRNOT00000115234 | 176,108 | -3,5227 | 0,340539 | 3,73E-27 | 4,38E-06 | protein_coding | Zfp672 | zinc finger protein 672 [Source:RGD Symbol;Acc:1359094] |
| ENSRNOT00000084505 | 376,2549 | -3,53079 | 0,224142 | 6,01E-58 | 5,83E-15 | protein_coding | Cab39l | calcium binding protein 39-like [Source:RGD Symbol;Acc:1308945] |
| ENSRNOT00000022063 | 2460,972 | -3,53815 | 0,200818 | 5,53E-71 | 5,83E-15 | protein_coding | Ift140 | intraflagellar transport 140 [Source:RGD Symbol;Acc:2318759] |
| ENSRNOT00000074131 | 453,5246 | -3,5398 | 0,208959 | 4,96E-66 | 5,83E-15 | protein_coding | Pnkd | PNKD metallo-beta-lactamase domain containing [Source:RGD Symbol;Acc:2300155] |
| ENSRNOT00000027297 | 118,3922 | -3,54357 | 0,308713 | 1,76E-32 | 1,92E-07 | protein_coding | Ahsp | alpha hemoglobin stabilizing protein [Source:RGD Symbol;Acc:1306725] |
| ENSRNOT00000048146 | 460,2807 | -3,54539 | 0,277867 | 3,52E-39 | 6,33E-14 | protein_coding | Tex56p | testis expressed 56 [Source:RGD Symbol;Acc:1307537] |
| ENSRNOT00000021063 | 686,621 | -3,55132 | 0,23731 | 1,11E-52 | 5,83E-15 | protein_coding | Spata1 | spermatogenesis associated 1 [Source:RGD Symbol;Acc:1359500] |
| ENSRNOT00000011084 | 3112,08 | -3,55315 | 0,220214 | 4,34E-60 | 5,83E-15 | protein_coding | Shc4 | SHC adaptor protein 4 [Source:RGD Symbol;Acc:1583644] |
| ENSRNOT00000008034 | 319,0015 | -3,55704 | 0,226106 | 2,3E-57 | 5,83E-15 | protein_coding | Slc2a6 | solute carrier family 2 member 6 [Source:RGD Symbol;Acc:1309317] |
| **ENSRNOT00000081355** | **541,297** | **-3,55785** | **0,25181** | **5,45E-47** | **1,48E-10** | **protein_coding** | **Tuba1a** | **tubulin, alpha 1A [Source:RGD Symbol;Acc:619717]** |
| ENSRNOT00000101364 | 283,9175 | -3,55875 | 0,319065 | 9,13E-31 | 2E-06 | protein_coding | Gm10135 | predicted gene 10135 [Source:RGD Symbol;Acc:150345068] |
| ENSRNOT00000108183 | 1844,373 | -3,57106 | 0,210409 | 2,17E-66 | 5,83E-15 | protein_coding | Gm17606 | predicted gene, 17606 [Source:RGD Symbol;Acc:150341735] |
| ENSRNOT00000084076 | 329,08 | -3,57375 | 0,290757 | 1,21E-36 | 8,29E-10 | protein_coding | Lig3 | DNA ligase 3 [Source:RGD Symbol;Acc:1309875] |
| ENSRNOT00000012404 | 10192,99 | -3,57392 | 0,290188 | 9,77E-37 | 1,36E-08 | protein_coding | Zwilch | zwilch kinetochore protein [Source:RGD Symbol;Acc:1583880] |
| ENSRNOT00000039593 | 351,66 | -3,57453 | 0,303769 | 8,86E-34 | 9,39E-07 | protein_coding | Msl2 | MSL complex subunit 2 [Source:RGD Symbol;Acc:1310355] |
| ENSRNOT00000023974 | 1103,638 | -3,57817 | 0,203982 | 1,65E-70 | 5,83E-15 | protein_coding | Mtr | 5-methyltetrahydrofolate-homocysteine methyltransferase [Source:RGD Symbol;Acc:621283] |
| ENSRNOT00000009944 | 3440,132 | -3,579 | 0,248221 | 6,06E-49 | 5,83E-15 | protein_coding | Cct6b | chaperonin containing TCP1 subunit 6B [Source:RGD Symbol;Acc:1359453] |
| ENSRNOT00000016208 | 443,6443 | -3,58688 | 0,258883 | 1,82E-45 | 5,28E-12 | protein_coding | Xrcc3 | X-ray repair cross complementing 3 [Source:RGD Symbol;Acc:1306849] |
| ENSRNOT00000020201 | 224,7152 | -3,58932 | 0,300807 | 6,57E-35 | 2,42E-08 | protein_coding | Gna14 | G protein subunit alpha 14 [Source:RGD Symbol;Acc:1308122] |
| ENSRNOT00000110443 | 222,4575 | -3,59494 | 0,301892 | 1,26E-34 | 7,24E-09 | protein_coding | Ddx39a | DExD-box helicase 39A [Source:RGD Symbol;Acc:619920] |
| ENSRNOT00000093978 | 153,2745 | -3,5981 | 0,384382 | 7,49E-23 | 0,001309 | protein_coding | Zfp27 | zinc finger protein 27 [Source:RGD Symbol;Acc:2318958] |
| ENSRNOT00000025224 | 110,9938 | -3,59949 | 0,371491 | 3,66E-24 | 1,26E-05 | protein_coding | AABR07028237.1 | |
| ENSRNOT00000061709 | 151,021 | -3,60192 | 0,318293 | 2,05E-31 | 5,38E-07 | protein_coding | RGD1565158 | similar to RIKEN cDNA 4921537P18 [Source:RGD Symbol;Acc:1565158] |
| ENSRNOT00000113907 | 949,6565 | -3,60528 | 0,215345 | 1,75E-64 | 5,83E-15 | protein_coding | Uqcc5 | ubiquinol-cytochrome c reductase complex assembly factor 5 [Source:RGD Symbol;Acc:1307583] |
| ENSRNOT00000022270 | 373,4642 | -3,60817 | 0,211453 | 4,84E-67 | 5,83E-15 | protein_coding | Dsg2 | desmoglein 2 [Source:RGD Symbol;Acc:1311143] |
| ENSRNOT00000109927 | 9511,762 | -3,61114 | 0,202763 | 1,16E-72 | 5,83E-15 | protein_coding | Rprd1b | regulation of nuclear pre-mRNA domain containing 1B [Source:RGD Symbol;Acc:1304782] |
| ENSRNOT00000095405 | 395,8547 | -3,61218 | 0,249652 | 2,9E-49 | 5,83E-15 | protein_coding | N6amt1 | N-6 adenine-specific DNA methyltransferase 1 [Source:RGD Symbol;Acc:1311843] |
| ENSRNOT00000023677 | 1437,368 | -3,61923 | 0,225361 | 1,54E-59 | 5,83E-15 | protein_coding | Pcsk7 | proprotein convertase subtilisin/kexin type 7 [Source:RGD Symbol;Acc:3275] |
| ENSRNOT00000014250 | 4664,755 | -3,62362 | 0,262337 | 3,64E-45 | 8,04E-13 | protein_coding | Syn1 | synapsin I [Source:RGD Symbol;Acc:3797] |
| ENSRNOT00000021216 | 350,2934 | -3,62986 | 0,29133 | 1,68E-37 | 5,05E-10 | protein_coding | Neurl2 | neuralized E3 ubiquitin protein ligase 2 [Source:RGD Symbol;Acc:1306409] |
| ENSRNOT00000019871 | 2721,958 | -3,6349 | 0,291563 | 1,38E-37 | 5,18E-10 | protein_coding | LOC100362400 | 60S ribosomal protein L13-like [Source:RGD Symbol;Acc:2318188] |
| ENSRNOT00000104232 | 295,3074 | -3,64023 | 0,299245 | 5,77E-36 | 3,97E-08 | protein_coding | LOC102554315 | zinc finger protein 596-like [Source:RGD Symbol;Acc:7747897] |
| ENSRNOT00000011027 | 385,7444 | -3,64255 | 0,363283 | 1,35E-25 | 0,001806 | protein_coding | Hoxb3 | homeo box B3 [Source:RGD Symbol;Acc:1310780] |
| ENSRNOT00000117186 | 788,9443 | -3,6481 | 0,217117 | 5,62E-65 | 5,83E-15 | protein_coding | Ndufaf7 | NADH:ubiquinone oxidoreductase complex assembly factor 7 [Source:RGD Symbol;Acc:1311578] |
| ENSRNOT00000013997 | 914,6601 | -3,64841 | 0,2177 | 1,08E-64 | 5,83E-15 | protein_coding | Psmc5 | proteasome 26S subunit, ATPase 5 [Source:RGD Symbol;Acc:708376] |
| ENSRNOT00000084372 | 1904,693 | -3,64852 | 0,227537 | 2,07E-59 | 5,83E-15 | protein_coding | Slc23a1 | solute carrier family 23 member 1 [Source:RGD Symbol;Acc:619875] |
| ENSRNOT00000071483 | 280,0986 | -3,64913 | 0,274178 | 2,51E-42 | 2,72E-14 | protein_coding | Ncbp2 | nuclear cap binding protein subunit 2 [Source:RGD Symbol;Acc:1596188] |
| ENSRNOT00000043384 | 966,0689 | -3,6586 | 0,362912 | 8,23E-26 | 8,49E-05 | protein_coding | Mcph1 | microcephalin 1 [Source:RGD Symbol;Acc:1560642] |
| ENSRNOT00000116539 | 291,1151 | -3,6594 | 0,233958 | 8,54E-57 | 5,83E-15 | protein_coding | Tkfc | triokinase and FMN cyclase [Source:RGD Symbol;Acc:1311026] |
| ENSRNOT00000030919 | 1258,017 | -3,66375 | 0,351654 | 2,49E-27 | 0,004124 | protein_coding | Fth1 | ferritin heavy chain 1 [Source:RGD Symbol;Acc:2635] |
| ENSRNOT00000051467 | 250,4075 | -3,66471 | 0,322945 | 1,11E-31 | 2,11E-07 | protein_coding | Dot1l | DOT1 like histone lysine methyltransferase [Source:RGD Symbol;Acc:1306644] |
| ENSRNOT00000020910 | 981,879 | -3,66946 | 0,207516 | 1,52E-71 | 5,83E-15 | protein_coding | Rftn2 | raftlin family member 2 [Source:RGD Symbol;Acc:1307304] |
| ENSRNOT00000098832 | 154,7559 | -3,67028 | 0,380018 | 4,6E-24 | 1,41E-05 | protein_coding | Zfp362 | zinc finger protein 362 [Source:RGD Symbol;Acc:1306498] |
| ENSRNOT00000117583 | 3381,755 | -3,67135 | 0,332511 | 2,68E-30 | 6,56E-05 | protein_coding | Hist1h4m | histone cluster 1, H4m [Source:RGD Symbol;Acc:1306905] |
| ENSRNOT00000112783 | 165,3517 | -3,67139 | 0,28136 | 5,22E-41 | 5,83E-15 | protein_coding | Tti2 | TELO2 interacting protein 2 [Source:RGD Symbol;Acc:1310414] |
| ENSRNOT00000113691 | 149,9859 | -3,67262 | 0,367331 | 1,71E-25 | 6,12E-07 | protein_coding | Rpp38 | ribonuclease P/MRP subunit p38 [Source:RGD Symbol;Acc:1307474] |
| ENSRNOT00000042161 | 2953,483 | -3,69415 | 0,165173 | 2,1E-111 | 5,83E-15 | protein_coding | LOC100362040 | Ac2-143-like [Source:RGD Symbol;Acc:2324563] |
| ENSRNOT00000067393 | 1739,372 | -3,70077 | 0,236122 | 3,81E-57 | 5,83E-15 | protein_coding | Asb3 | ankyrin repeat and SOCS box-containing 3 [Source:RGD Symbol;Acc:1308462] |
| ENSRNOT00000097990 | 390,9743 | -3,70162 | 0,255624 | 2,04E-49 | 5,83E-15 | protein_coding | Psmg2 | proteasome assembly chaperone 2 [Source:RGD Symbol;Acc:1304867] |
| ENSRNOT00000105403 | 586,6799 | -3,71016 | 0,253114 | 1,37E-50 | 5,83E-15 | protein_coding | Glt8d2 | glycosyltransferase 8 domain containing 2 [Source:RGD Symbol;Acc:1560432] |
| ENSRNOT00000106507 | 6241,453 | -3,71292 | 0,196439 | 3,49E-81 | 5,83E-15 | protein_coding | Ccdc162 | coiled-coil domain containing 162 [Source:RGD Symbol;Acc:7514220] |
| ENSRNOT00000014018 | 846,1892 | -3,71639 | 0,214514 | 5,03E-69 | 5,83E-15 | protein_coding | Pnma1 | PNMA family member 1 [Source:RGD Symbol;Acc:621230] |
| ENSRNOT00000028699 | 660,5193 | -3,73496 | 0,225921 | 4,45E-63 | 5,83E-15 | protein_coding | Cers2 | ceramide synthase 2 [Source:RGD Symbol;Acc:1310059] |
| ENSRNOT00000115879 | 261,3711 | -3,74033 | 0,296316 | 2,49E-38 | 5,5E-10 | protein_coding | Pdcd2 | programmed cell death 2 [Source:RGD Symbol;Acc:61887] |
| ENSRNOT00000031488 | 638,921 | -3,74738 | 0,191109 | 5,04E-87 | 5,83E-15 | protein_coding | Entrep2 | endosomal transmembrane epsin interactor 2 [Source:RGD Symbol;Acc:1592985] |
| ENSRNOT00000039252 | 3699,913 | -3,75358 | 0,204783 | 1,47E-76 | 5,83E-15 | protein_coding | Polr2b | RNA polymerase II subunit B [Source:RGD Symbol;Acc:1309477] |
| ENSRNOT00000096607 | 1299,754 | -3,75567 | 0,269441 | 5,96E-46 | 3,1E-12 | protein_coding | Opa3 | outer mitochondrial membrane lipid metabolism regulator OPA3 [Source:RGD Symbol;Acc:1309147] |
| ENSRNOT00000111808 | 185,0653 | -3,76012 | 0,302095 | 2,69E-37 | 9,83E-14 | protein_coding | Msto1 | misato mitochondrial distribution and morphology regulator 1 [Source:RGD Symbol;Acc:1306110] |
| ENSRNOT00000102408 | 4109,435 | -3,76358 | 0,18025 | 5,2E-98 | 5,83E-15 | protein_coding | Cdk4 | cyclin-dependent kinase 4 [Source:RGD Symbol;Acc:621120] |
| ENSRNOT00000105114 | 1952,423 | -3,76378 | 0,266408 | 6,4E-47 | 5,83E-15 | protein_coding | Phykpl | 5-phosphohydroxy-L-lysine phospho-lyase [Source:RGD Symbol;Acc:2293818] |
| ENSRNOT00000079442 | 515,4589 | -3,76404 | 0,291378 | 5,89E-40 | 2,59E-08 | protein_coding | Prr33 | proline rich 33 [Source:RGD Symbol;Acc:1592568] |
| ENSRNOT00000035815 | 418,9588 | -3,76455 | 0,218187 | 2,79E-68 | 5,83E-15 | protein_coding | RGD1309106 | similar to hypothetical protein [Source:RGD Symbol;Acc:1309106] |
| ENSRNOT00000079745 | 102,1956 | -3,76904 | 0,383487 | 7,32E-25 | 7,37E-06 | protein_coding | Ccna2 | cyclin A2 [Source:RGD Symbol;Acc:621059] |
| ENSRNOT00000077228 | 141,2344 | -3,77107 | 0,327226 | 1,02E-32 | 3,57E-12 | protein_coding | Ccdc51 | coiled-coil domain containing 51 [Source:RGD Symbol;Acc:1311466] |
| ENSRNOT00000001712 | 561,5297 | -3,77188 | 0,194431 | 2,82E-85 | 5,83E-15 | protein_coding | Hvcn1 | hydrogen voltage-gated channel 1 [Source:RGD Symbol;Acc:1310788] |
| ENSRNOT00000026321 | 563,5142 | -3,77334 | 0,299645 | 4,09E-38 | 9,53E-11 | protein_coding | Zbtb3 | zinc finger and BTB domain containing 3 [Source:RGD Symbol;Acc:1561140] |
| ENSRNOT00000101637 | 218,1058 | -3,79832 | 0,277304 | 1,51E-44 | 5,83E-15 | protein_coding | Mrpl18 | mitochondrial ribosomal protein L18 [Source:RGD Symbol;Acc:1306387] |
| ENSRNOT00000040672 | 421,2446 | -3,80149 | 0,295884 | 1,09E-39 | 1,2E-10 | protein_coding | C1h11orf42 | similar to human chromosome 11 open reading frame 42 [Source:RGD Symbol;Acc:1561034] |
| ENSRNOT00000067150 | 143,1407 | -3,80421 | 0,320444 | 3,27E-34 | 2,22E-14 | protein_coding | Gmpr2 | guanosine monophosphate reductase 2 [Source:RGD Symbol;Acc:628875] |
| ENSRNOT00000101419 | 621,9355 | -3,80518 | 0,220099 | 1,33E-68 | 5,83E-15 | protein_coding | Ifi35 | interferon-induced protein 35 [Source:RGD Symbol;Acc:1304553] |
| ENSRNOT00000020297 | 285,3117 | -3,80918 | 0,313305 | 6,9E-36 | 5,17E-09 | protein_coding | Lrrc39 | leucine rich repeat containing 39 [Source:RGD Symbol;Acc:1585106] |
| ENSRNOT00000010730 | 469,7892 | -3,81195 | 0,267879 | 8,07E-48 | 5,83E-15 | protein_coding | Sync | syncoilin, intermediate filament protein [Source:RGD Symbol;Acc:1307970] |
| ENSRNOT00000031385 | 685,373 | -3,81543 | 0,204709 | 4,64E-79 | 5,83E-15 | protein_coding | Ccdc125 | coiled-coil domain containing 125 [Source:RGD Symbol;Acc:1561673] |
| ENSRNOT00000103139 | 612,5576 | -3,82357 | 0,364317 | 8,71E-28 | 0,003873 | protein_coding | Tmprss6 | transmembrane serine protease 6 [Source:RGD Symbol;Acc:1307138] |
| ENSRNOT00000113188 | 519,3869 | -3,83555 | 0,222591 | 4,86E-68 | 5,83E-15 | protein_coding | Zbtb3 | zinc finger and BTB domain containing 3 [Source:RGD Symbol;Acc:1561140] |
| ENSRNOT00000104939 | 284,0289 | -3,84756 | 0,296103 | 1,39E-40 | 2,37E-13 | protein_coding | Tfpt | TCF3 fusion partner [Source:RGD Symbol;Acc:620839] |
| ENSRNOT00000108223 | 133,1628 | -3,88151 | 0,362033 | 1,24E-28 | 9,68E-06 | protein_coding | Tmem256 | transmembrane protein 256 [Source:RGD Symbol;Acc:1563438] |
| ENSRNOT00000095083 | 570,7894 | -3,88721 | 0,211193 | 3,99E-77 | 5,83E-15 | protein_coding | Usp42 | ubiquitin specific peptidase 42 [Source:RGD Symbol;Acc:1305231] |
| ENSRNOT00000065600 | 117,0685 | -3,89023 | 0,363228 | 1,49E-28 | 2,41E-09 | protein_coding | Ttc3 | tetratricopeptide repeat domain 3 [Source:RGD Symbol;Acc:1308654] |
| ENSRNOT00000097794 | 144,5841 | -3,89143 | 0,334181 | 2,87E-33 | 5,91E-12 | protein_coding | LOC691519 | similar to ankyrin repeat domain 26 [Source:RGD Symbol;Acc:1582795] |
| ENSRNOT00000100853 | 331,6055 | -3,89422 | 0,379633 | 8,7E-27 | 0,000396 | protein_coding | Tmem200b | transmembrane protein 200B [Source:RGD Symbol;Acc:1585251] |
| ENSRNOT00000063873 | 229,4687 | -3,90053 | 0,292851 | 1,88E-42 | 5,84E-14 | protein_coding | Tm6sf1 | transmembrane 6 superfamily member 1 [Source:RGD Symbol;Acc:1307978] |
| ENSRNOT00000006325 | 249,3066 | -3,90194 | 0,309704 | 3,51E-38 | 5,27E-11 | protein_coding | Pp2d1 | protein phosphatase 2C-like domain containing 1 [Source:RGD Symbol;Acc:1564811] |
| ENSRNOT00000116448 | 249,459 | -3,90462 | 0,276407 | 7,37E-47 | 5,83E-15 | protein_coding | Sirt3 | sirtuin 3 [Source:RGD Symbol;Acc:1308374] |
| ENSRNOT00000115817 | 114,9102 | -3,90806 | 0,36295 | 6,52E-29 | 2,72E-08 | protein_coding | Nfya | nuclear transcription factor Y subunit alpha [Source:RGD Symbol;Acc:70976] |
| ENSRNOT00000101050 | 251,5202 | -3,90946 | 0,292167 | 5,72E-43 | 5,83E-15 | protein_coding | Tbc1d31 | TBC1 domain family, member 31 [Source:RGD Symbol;Acc:1587370] |
| ENSRNOT00000026539 | 143,2749 | -3,91675 | 0,319775 | 2,18E-36 | 6,15E-12 | protein_coding | Gng3 | G protein subunit gamma 3 [Source:RGD Symbol;Acc:620805] |
| ENSRNOT00000103264 | 186,6052 | -3,94088 | 0,347322 | 1,24E-31 | 4,43E-07 | protein_coding | Atg16l2 | autophagy related 16-like 2 [Source:RGD Symbol;Acc:1311400] |
| ENSRNOT00000104557 | 1564,661 | -3,94821 | 0,205546 | 1,08E-83 | 5,83E-15 | protein_coding | Pnrc2 | proline-rich nuclear receptor coactivator 2 [Source:RGD Symbol;Acc:1642418] |
| ENSRNOT00000033784 | 231,7852 | -3,94975 | 0,350527 | 3,12E-31 | 3,62E-05 | protein_coding | Kif20a | kinesin family member 20A [Source:RGD Symbol;Acc:1307695] |
| ENSRNOT00000119675 | 221,7252 | -3,95243 | 0,342033 | 8,38E-33 | 4,3E-12 | protein_coding | Hsf2 | heat shock transcription factor 2 [Source:RGD Symbol;Acc:68395] |
| ENSRNOT00000084824 | 4121,418 | -3,95391 | 0,448369 | 1,36E-20 | 0,007102 | protein_coding | Sgf29 | SAGA complex associated factor 29 [Source:RGD Symbol;Acc:1310609] |
| ENSRNOT00000041866 | 1820,769 | -3,95679 | 0,279706 | 4,49E-47 | 1,69E-14 | protein_coding | AABR07072400.2 | |
| **ENSRNOT00000021405** | **2366,993** | **-3,96613** | **0,277465** | **5,62E-48** | **2,67E-11** | **protein_coding** | **Myo7b** | **myosin VIIb [Source:RGD Symbol;Acc:1561153]** |
| ENSRNOT00000020660 | 124,1405 | -3,96992 | 0,398236 | 2,21E-25 | 1,68E-06 | protein_coding | Cfap157 | cilia and flagella associated protein 157 [Source:RGD Symbol;Acc:1560002] |
| ENSRNOT00000113995 | 265,3749 | -3,97113 | 0,333282 | 1,04E-34 | 5,95E-11 | protein_coding | Psmc1 | proteasome 26S subunit, ATPase 1 [Source:RGD Symbol;Acc:621097] |
| ENSRNOT00000087113 | 539,5589 | -3,97378 | 0,211025 | 1,57E-80 | 5,83E-15 | protein_coding | Clec12a | C-type lectin domain family 12, member A [Source:RGD Symbol;Acc:1592991] |
| ENSRNOT00000015238 | 119,8241 | -3,99544 | 0,383232 | 2,21E-27 | 4,57E-06 | protein_coding | Usp50 | ubiquitin specific peptidase 50 [Source:RGD Symbol;Acc:1564751] |
| ENSRNOT00000116702 | 125,3003 | -4,00007 | 0,344076 | 4,49E-33 | 2,02E-11 | protein_coding | Setd1b | SET domain containing 1B, histone lysine methyltransferase [Source:RGD Symbol;Acc:2323325] |
| ENSRNOT00000037690 | 839,9174 | -4,00075 | 0,189857 | 1E-99 | 5,83E-15 | protein_coding | Pkd2l2 | polycystin 2 like 2, transient receptor potential cation channel [Source:RGD Symbol;Acc:1310272] |
| ENSRNOT00000079495 | 103,5272 | -4,00109 | 0,337273 | 3,63E-34 | 5,83E-15 | protein_coding | Rpe | ribulose-5-phosphate-3-epimerase [Source:RGD Symbol;Acc:1564890] |
| ENSRNOT00000099804 | 902,797 | -4,01091 | 0,226969 | 2,66E-71 | 5,83E-15 | protein_coding | Dusp18 | dual specificity phosphatase 18 [Source:RGD Symbol;Acc:1306929] |
| ENSRNOT00000026778 | 2902,472 | -4,01311 | 0,289715 | 2,25E-45 | 4,3E-14 | protein_coding | Paf1 | PAF1 homolog, Paf1/RNA polymerase II complex component [Source:RGD Symbol;Acc:1306219] |
| ENSRNOT00000051824 | 120,7208 | -4,01835 | 0,342765 | 1,23E-33 | 6,6E-13 | protein_coding | LOC498231 | LRRGT00144 [Source:RGD Symbol;Acc:1561566] |
| ENSRNOT00000077345 | 419,4241 | -4,02718 | 0,238321 | 1,39E-65 | 5,83E-15 | protein_coding | Sdccag8 | SHH signaling and ciliogenesis regulator SDCCAG8 [Source:RGD Symbol;Acc:727781] |
| ENSRNOT00000113178 | 254,9458 | -4,05482 | 0,269645 | 4,4E-53 | 5,83E-15 | protein_coding | Pyroxd1 | pyridine nucleotide-disulphide oxidoreductase domain 1 [Source:RGD Symbol;Acc:1303253] |
| ENSRNOT00000094074 | 265,9296 | -4,07738 | 0,283223 | 1,07E-48 | 5,83E-15 | protein_coding | Acp4 | acid phosphatase 4 [Source:RGD Symbol;Acc:1308270] |
| ENSRNOT00000113908 | 442,195 | -4,08016 | 0,249342 | 9,05E-62 | 5,83E-15 | protein_coding | Gyg1 | glycogenin 1 [Source:RGD Symbol;Acc:621785] |
| ENSRNOT00000119815 | 546,4668 | -4,08109 | 0,24681 | 2,3E-63 | 5,83E-15 | protein_coding | Gm17720 | predicted gene, 17720 [Source:RGD Symbol;Acc:150342637] |
| ENSRNOT00000095468 | 2211,434 | -4,08921 | 0,313018 | 3,27E-41 | 4,9E-05 | protein_coding | Cbr4 | carbonyl reductase 4 [Source:RGD Symbol;Acc:727826] |
| ENSRNOT00000104846 | 646,8907 | -4,09085 | 0,316496 | 4,89E-40 | 3,21E-09 | protein_coding | Gpr17 | G protein-coupled receptor 17 [Source:RGD Symbol;Acc:1589785] |
| ENSRNOT00000101981 | 261,5642 | -4,10311 | 0,280324 | 2,15E-50 | 5,83E-15 | protein_coding | Ankzf1 | ankyrin repeat and zinc finger peptidyl tRNA hydrolase 1 [Source:RGD Symbol;Acc:1359242] |
| ENSRNOT00000085891 | 1002,406 | -4,11127 | 0,252026 | 2,04E-61 | 5,83E-15 | protein_coding | Phaf1 | phagosome assembly factor 1 [Source:RGD Symbol;Acc:621098] |
| ENSRNOT00000046512 | 319,297 | -4,11821 | 0,254985 | 2E-60 | 5,83E-15 | protein_coding | Serpinc1 | serpin family C member 1 [Source:RGD Symbol;Acc:1307404] |
| ENSRNOT00000033234 | 222,4991 | -4,14215 | 0,357104 | 5,27E-33 | 3,4E-08 | protein_coding | Ankrd53 | ankyrin repeat domain 53 [Source:RGD Symbol;Acc:1561200] |
| ENSRNOT00000101859 | 2269,433 | -4,15504 | 0,29764 | 3,32E-46 | 1,97E-06 | protein_coding | Acox3 | acyl-CoA oxidase 3, pristanoyl [Source:RGD Symbol;Acc:69245] |
| ENSRNOT00000115454 | 261,0186 | -4,16114 | 0,364911 | 5,23E-32 | 2,26E-08 | protein_coding | LOC685989 | hypothetical protein LOC685989 [Source:RGD Symbol;Acc:1587786] |
| ENSRNOT00000104618 | 397,5481 | -4,16688 | 0,294042 | 3,01E-47 | 5,83E-15 | protein_coding | Cfl1 | cofilin 1 [Source:RGD Symbol;Acc:69285] |
| ENSRNOT00000020889 | 465,7914 | -4,16698 | 0,254908 | 1,32E-61 | 5,83E-15 | protein_coding | Wdr27 | WD repeat domain 27 [Source:RGD Symbol;Acc:1309633] |
| ENSRNOT00000076100 | 492,9261 | -4,17059 | 0,256009 | 3,04E-61 | 5,83E-15 | protein_coding | Rrp8 | ribosomal RNA processing 8 [Source:RGD Symbol;Acc:1308302] |
| ENSRNOT00000116546 | 367,7973 | -4,18028 | 0,295441 | 3,74E-47 | 5,83E-15 | protein_coding | Snx14 | sorting nexin 14 [Source:RGD Symbol;Acc:1310921] |
| ENSRNOT00000033126 | 947,4415 | -4,18729 | 0,194584 | 1E-103 | 5,83E-15 | protein_coding | Fbxl2 | F-box and leucine-rich repeat protein 2 [Source:RGD Symbol;Acc:1562243] |
| **ENSRNOT00000008387** | **121,5653** | **-4,19393** | **0,336237** | **1,1E-37** | **1,69E-14** | **protein_coding** | **Chrm5** | **cholinergic receptor, muscarinic 5 [Source:RGD Symbol;Acc:620027]** |
| ENSRNOT00000002447 | 1121,632 | -4,1983 | 0,331843 | 1,75E-38 | 9,48E-09 | protein_coding | AC133403.1 | |
| ENSRNOT00000012296 | 1032,591 | -4,20892 | 0,408571 | 6,25E-27 | 1,22E-05 | protein_coding | Ifi27 | interferon, alpha-inducible protein 27 [Source:RGD Symbol;Acc:69404] |
| ENSRNOT00000103292 | 3569,491 | -4,21547 | 0,205455 | 8,3E-95 | 5,83E-15 | protein_coding | Enoph1 | enolase-phosphatase 1 [Source:RGD Symbol;Acc:1309016] |
| ENSRNOT00000102558 | 102,8471 | -4,21784 | 0,377396 | 8,01E-31 | 2,52E-10 | protein_coding | Pou2af3 | POU class 2 homeobox associating factor 3 [Source:RGD Symbol;Acc:1563866] |
| ENSRNOT00000046659 | 115,5149 | -4,23777 | 0,363657 | 2,41E-33 | 5,59E-11 | protein_coding | Frmd5 | FERM domain containing 5 [Source:RGD Symbol;Acc:1561554] |
| ENSRNOT00000110124 | 356,217 | -4,2465 | 0,259271 | 3,16E-62 | 5,83E-15 | protein_coding | Sdcbp | syndecan binding protein [Source:RGD Symbol;Acc:621497] |
| ENSRNOT00000104515 | 220,8717 | -4,25916 | 0,270571 | 1,14E-57 | 5,83E-15 | protein_coding | Rbis | ribosomal biogenesis factor [Source:RGD Symbol;Acc:1565641] |
| ENSRNOT00000082469 | 8647,414 | -4,2899 | 0,26587 | 3,19E-60 | 5,83E-15 | protein_coding | Dcaf8 | DDB1 and CUL4 associated factor 8 [Source:RGD Symbol;Acc:1308513] |
| ENSRNOT00000111053 | 1009,124 | -4,30105 | 0,306982 | 1,37E-46 | 1,11E-12 | protein_coding | Agbl5 | AGBL carboxypeptidase 5 [Source:RGD Symbol;Acc:1598311] |
| ENSRNOT00000098376 | 393,6288 | -4,30125 | 0,254165 | 8,55E-66 | 5,83E-15 | protein_coding | Znrd1as1 | ZNRD1 antisense RNA 1 [Source:RGD Symbol;Acc:1564067] |
| ENSRNOT00000111037 | 135,5887 | -4,3063 | 0,348692 | 8,06E-37 | 3,91E-11 | protein_coding | Gm17651 | predicted gene, 17651 [Source:RGD Symbol;Acc:150342749] |
| ENSRNOT00000056169 | 12948,18 | -4,31422 | 0,196455 | 1E-107 | 5,83E-15 | protein_coding | Kdelr3 | KDEL endoplasmic reticulum protein retention receptor 3 [Source:RGD Symbol;Acc:1311536] |
| ENSRNOT00000076985 | 877,2986 | -4,33256 | 0,23997 | 1,54E-74 | 5,83E-15 | protein_coding | Zbtb37 | zinc finger and BTB domain containing 37 [Source:RGD Symbol;Acc:1308731] |
| ENSRNOT00000109399 | 312,2338 | -4,33409 | 0,360415 | 3,27E-35 | 4,39E-08 | protein_coding | Dnai3 | dynein axonemal intermediate chain 3 [Source:RGD Symbol;Acc:1563105] |
| ENSRNOT00000010712 | 425,356 | -4,33739 | 0,495792 | 2,55E-20 | 0,000979 | protein_coding | Fos | Fos proto-oncogene, AP-1 transcription factor subunit [Source:RGD Symbol;Acc:2626] |
| ENSRNOT00000097872 | 1294,264 | -4,36081 | 0,219243 | 2,07E-89 | 5,83E-15 | protein_coding | Fgf16 | fibroblast growth factor 16 [Source:RGD Symbol;Acc:71052] |
| ENSRNOT00000096135 | 114,9026 | -4,38558 | 0,421473 | 3,12E-27 | 1,08E-05 | protein_coding | Rnf214 | ring finger protein 214 [Source:RGD Symbol;Acc:1307500] |
| ENSRNOT00000013896 | 418,1276 | -4,38621 | 0,272017 | 6,95E-60 | 5,83E-15 | protein_coding | Serpina3c | serine (or cysteine) proteinase inhibitor, clade A, member 3C [Source:RGD Symbol;Acc:2972] |
| ENSRNOT00000049820 | 839,5121 | -4,40582 | 0,277253 | 1,38E-58 | 5,83E-15 | protein_coding | Leap2 | liver enriched antimicrobial peptide 2 [Source:RGD Symbol;Acc:1561235] |
| ENSRNOT00000009744 | 704,3959 | -4,41325 | 0,332418 | 4,45E-42 | 4,74E-13 | protein_coding | Clvs1 | clavesin 1 [Source:RGD Symbol;Acc:1564200] |
| ENSRNOT00000115194 | 270,0795 | -4,53842 | 0,293823 | 8,62E-56 | 5,83E-15 | protein_coding | Nell2 | neural EGFL like 2 [Source:RGD Symbol;Acc:620999] |
| ENSRNOT00000046060 | 3251,662 | -4,55833 | 0,270185 | 2,21E-65 | 5,83E-15 | protein_coding | LOC304725 | similar to contactin associated protein-like 5 isoform 1 [Source:RGD Symbol;Acc:1592077] |
| ENSRNOT00000027208 | 1827,32 | -4,59031 | 0,311768 | 5,02E-51 | 1,25E-12 | protein_coding | Tmem198 | transmembrane protein 198 [Source:RGD Symbol;Acc:2319152] |
| ENSRNOT00000079582 | 903,7934 | -4,6008 | 0,268166 | 1,23E-67 | 5,83E-15 | protein_coding | AABR07006025.2 | |
| ENSRNOT00000106246 | 174,2822 | -4,61253 | 0,405398 | 8,66E-32 | 8,7E-09 | protein_coding | Atxn7 | ataxin 7 [Source:RGD Symbol;Acc:1562692] |
| ENSRNOT00000016317 | 213,3683 | -4,61562 | 0,306093 | 2,14E-53 | 5,83E-15 | protein_coding | Masp2 | MBL associated serine protease 2 [Source:RGD Symbol;Acc:620214] |
| ENSRNOT00000075091 | 199,3574 | -4,6213 | 0,322742 | 2,1E-48 | 5,83E-15 | protein_coding | LOC100366030 | rCG37858-like [Source:RGD Symbol;Acc:2324613] |
| ENSRNOT00000116207 | 628,2523 | -4,64067 | 0,314089 | 2,75E-51 | 1,54E-11 | protein_coding | Olr1585 | olfactory receptor 1585 [Source:RGD Symbol;Acc:1333297] |
| ENSRNOT00000020600 | 304,2835 | -4,6701 | 0,46889 | 2,5E-25 | 0,000201 | protein_coding | Pigf | phosphatidylinositol glycan anchor biosynthesis, class F [Source:RGD Symbol;Acc:1584658] |
| ENSRNOT00000082963 | 592,6314 | -4,71194 | 0,291229 | 1,05E-60 | 5,83E-15 | protein_coding | Tpgs2 | tubulin polyglutamylase complex subunit 2 [Source:RGD Symbol;Acc:1310571] |
| ENSRNOT00000080696 | 388,2455 | -4,72142 | 0,343433 | 7,46E-45 | 2,84E-13 | protein_coding | AABR07025896.1 | |
| ENSRNOT00000113192 | 182,4041 | -4,74228 | 0,365431 | 1,87E-40 | 2,15E-13 | protein_coding | Apeg3 | antisense paternally expressed gene 3 [Source:RGD Symbol;Acc:1563921] |
| ENSRNOT00000012381 | 161,3806 | -4,74493 | 0,441693 | 8,19E-29 | 7,84E-05 | protein_coding | Rec114 | REC114 meiotic recombination protein [Source:RGD Symbol;Acc:1311874] |
| ENSRNOT00000036823 | 2282,101 | -4,76305 | 0,185443 | 8,7E-146 | 5,83E-15 | protein_coding | Ell3 | elongation factor for RNA polymerase II 3 [Source:RGD Symbol;Acc:1309917] |
| ENSRNOT00000106111 | 12146,86 | -4,79185 | 0,331955 | 4,3E-49 | 1,21E-10 | protein_coding | Cmss1 | cms1 ribosomal small subunit homolog [Source:RGD Symbol;Acc:1309437] |
| ENSRNOT00000011820 | 459,7059 | -4,80614 | 0,545966 | 1,49E-20 | 0,008294 | protein_coding | Tbc1d21 | TBC1 domain family, member 21 [Source:RGD Symbol;Acc:1310243] |
| ENSRNOT00000117088 | 223,1003 | -4,81231 | 0,334199 | 8,35E-49 | 5,83E-15 | protein_coding | Cfl1 | cofilin 1 [Source:RGD Symbol;Acc:69285] |
| ENSRNOT00000021670 | 250,0336 | -4,82155 | 0,455177 | 3,49E-28 | 0,000766 | protein_coding | Gpr162 | G protein-coupled receptor 162 [Source:RGD Symbol;Acc:1307437] |
| ENSRNOT00000099617 | 125,1106 | -4,90696 | 0,38604 | 8,82E-39 | 5,83E-15 | protein_coding | Wdr20 | WD repeat domain 20 [Source:RGD Symbol;Acc:1306499] |
| ENSRNOT00000074533 | 213,5083 | -4,94152 | 0,437153 | 2,26E-31 | 0,000383 | protein_coding | Ggt1 | gamma-glutamyltransferase 1 [Source:RGD Symbol;Acc:2683] |
| ENSRNOT00000009199 | 1197,008 | -4,94227 | 0,35798 | 4,43E-45 | 8,58E-08 | protein_coding | Pced1b | PC-esterase domain containing 1B [Source:RGD Symbol;Acc:1561028] |
| ENSRNOT00000043611 | 189,6464 | -4,95395 | 0,336804 | 7,81E-51 | 5,83E-15 | protein_coding | Slc24a5 | solute carrier family 24 member 5 [Source:RGD Symbol;Acc:1310565] |
| ENSRNOT00000095778 | 1690,564 | -5,03196 | 0,268125 | 5,37E-80 | 5,83E-15 | protein_coding | Mtrex | Mtr4 exosome RNA helicase [Source:RGD Symbol;Acc:1305984] |
| ENSRNOT00000112113 | 170,1947 | -5,03763 | 0,366812 | 1,16E-44 | 5,83E-15 | protein_coding | Smc4 | structural maintenance of chromosomes 4 [Source:RGD Symbol;Acc:1306680] |
| ENSRNOT00000093261 | 55107,65 | -5,11457 | 0,39944 | 2,05E-39 | 9,29E-07 | protein_coding | Cenpp | centromere protein P [Source:RGD Symbol;Acc:1589530] |
| ENSRNOT00000051338 | 2874,258 | -5,28736 | 0,286514 | 1,25E-77 | 5,83E-15 | protein_coding | Inpp5d | inositol polyphosphate-5-phosphatase D [Source:RGD Symbol;Acc:2914] |
| ENSRNOT00000030413 | 145,2261 | -5,30786 | 0,410148 | 3,97E-40 | 5,83E-15 | protein_coding | AC107446.1 | Sproutin [Source:RGD Symbol;Acc:15003554] |
| ENSRNOT00000096112 | 2745,397 | -5,32084 | 0,41743 | 4,4E-39 | 7,64E-07 | protein_coding | Rbbp4 | RB binding protein 4, chromatin remodeling factor [Source:RGD Symbol;Acc:1593768] |
| ENSRNOT00000043327 | 1545,455 | -5,33886 | 0,40735 | 1,65E-41 | 1,53E-06 | protein_coding | AABR07030544.1 | |
| ENSRNOT00000067997 | 701,7231 | -5,48873 | 0,383926 | 4,65E-48 | 2,72E-14 | protein_coding | LOC100360491 | 60S ribosomal protein L13-like [Source:RGD Symbol;Acc:2322065] |
| ENSRNOT00000104112 | 210,9143 | -5,50332 | 0,398219 | 2,91E-45 | 5,83E-15 | protein_coding | Etfdh | electron transfer flavoprotein dehydrogenase [Source:RGD Symbol;Acc:735052] |
| ENSRNOT00000050949 | 109,7979 | -5,56438 | 0,485832 | 2,9E-32 | 1,69E-14 | protein_coding | Bmp8b | bone morphogenetic protein 8b [Source:RGD Symbol;Acc:1591873] |
| ENSRNOT00000095403 | 4233,483 | -6,00451 | 0,279559 | 2,9E-103 | 5,83E-15 | protein_coding | Fancc | FA complementation group C [Source:RGD Symbol;Acc:2593] |
| ENSRNOT00000043036 | 224,2974 | -6,44848 | 0,482883 | 9,29E-43 | 5,83E-15 | protein_coding | Gabrp | gamma-aminobutyric acid type A receptor subunit pi [Source:RGD Symbol;Acc:620532] |
| ENSRNOT00000096004 | 127,7954 | -6,97098 | 0,662254 | 6,19E-28 | 5,83E-15 | protein_coding | Cnn1 | calponin 1 [Source:RGD Symbol;Acc:621883] |
| ENSRNOT00000111956 | 274,0346 | -7,09874 | 0,661109 | 9,18E-29 | 2,07E-06 | protein_coding | Kctd16 | potassium channel tetramerization domain containing 16 [Source:RGD Symbol;Acc:1559856] |
| ENSRNOT00000036777 | 224,7623 | -7,55197 | 0,792495 | 2,25E-23 | 0,002299 | protein_coding | Rtp2 | receptor (chemosensory) transporter protein 2 [Source:RGD Symbol;Acc:1565722] |
